# Topology-Based Detection and Tracking of Deadlocks Reveal Aging of Active Ring Melts

**DOI:** 10.1021/acsmacrolett.3c00567

**Published:** 2024-01-10

**Authors:** Cristian Micheletti, Iurii Chubak, Enzo Orlandini, Jan Smrek

**Affiliations:** † Scuola Internazionale Superiore di Studi Avanzati (SISSA), Via Bonomea 265, I-34136 Trieste, Italy; ‡ Sorbonne Université CNRS, Physico-Chimie des électrolytes et Nanosystèmes Interfaciaux, F-75005 Paris, France; § Università degli studi di Padova, Dipartimento di Fisica “G. Galilei”, Via Marzolo 8, I-35100 Padova, Italy; ∥ Faculty of Physics, University of Vienna, Boltzmanngasse 5, A-1090 Vienna, Austria

## Abstract

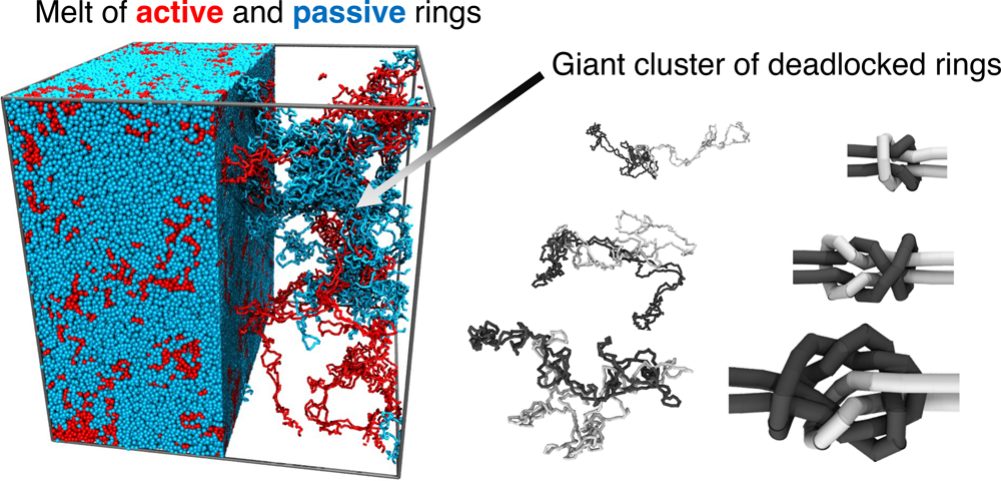

Connecting the viscoelastic behavior of stressed ring melts to the various forms of entanglement that can emerge in such systems is still an open challenge. Here, we consider active ring melts, where stress is generated internally, and introduce a topology-based method to detect and track consequential forms of ring entanglements, namely, deadlocks. We demonstrate that, as stress accumulates, more and more rings are co-opted in a growing web of deadlocks that entrap many other rings by threading, bringing the system to a standstill. The method ought to help the study of topological aging in more general polymer contexts.

The viscoelastic response of densely packed ring polymers is a major unsolved problem in soft matter physics. Unlike linear or branched chains,^1−11^ uncatenated rings in melts are compact fractals^12−14^ and, thus, feature unique relaxation modes,^15−19^ resulting in nonconventional linear stress–relaxation responses.^20−22^ Outside the linear regime, ring melts subject to external stresses, such as elongational flows, are prone to form not only threadings, but also interlocked superstructures,^23,24^ where several rings latch onto other ones, exhibiting a nonmonotonic response with the extensional rate.^23^ In contrast, the monotonic shear thinning of the melt and the behavior of the ring glass under tensile loading are similar to linear melts, suggesting no superstructures arise in these cases.^25−27^

Anomalous viscoelasticity can emerge in melts not only due to external perturbations, but also from internal stresses. Prototypic systems are blends of standard (passive) rings and active ones with a subset of monomers at higher-than-ambient temperature.^28^ These systems are inspired by chromatin fibers, whose active fluctuations have an effective temperature twice the ambient one^29,30^ and, prospectively, chains of flexibly linked colloids^31^ with addressable active segments.^32−34^ Ring mixtures with active components have been previously termed active topological glasses (ATG)^28,35^ because random initial fluid states evolve toward dynamically arrested ones. The degree of ATG “vitrification” has been found to correlate with the emergence of long-lived ring threadings,^28,35^ thus establishing an analogy with viscosity thickening in externally stressed ring melts in elongational flows. However, a mechanistic understanding of the connection between ring threadings and anomalous dynamics due to deadlocked superstructures in stressed melts is still lacking, mostly due to the absence of a general identification method for the deadlocks. Such a method would help address fundamental yet unexplored questions, such as the following: What types of deadlocks can generally emerge in stressed ring melts? Are such states already present in equilibrium? How do they evolve when rings are under stress? How are deadlocks connected to observed dynamical arrest?

Here, we address these questions with a novel, direct, and general method for detecting supramolecular deadlocks. We harness the scheme to track the microscopic evolution of interlockings in ATGs with hundreds of active and passive rings. The results expose the topological aging of the system and its microscopic bases for the first time.

The active ring melt that we considered consisted of *N* = 1600 unknotted rings, each of 400 beads, packed in a periodic cubic simulation box at the monomer density ρ = 0.85σ^–3^, with σ being the nominal bead diameter. The number of active rings, *m*, varied from 10 to 1600. Following the Kremer and Grest polymer model,^36,37^ the chain connectivity of the rings was provided by a FENE potential. Excluded volume interactions of intra- and inter-ring pairs of monomers were treated with a truncated and shifted Lennard-Jones potential. The combined FENE and LJ interactions disallow chain crossings, preventing rings from becoming knotted or linked. The system was evolved with Langevin dynamics simulations, integrated with the LAMMPS package.^38^ The damping parameter was set to 1.5τ, where τ is the characteristic MD time, and all beads were thermostated at unit temperature, *T* = 1, except for a 50-bead long stretch in each active ring, thermostated at *T*_a_ = 3*T*. This combination of activity and dissipation yields an effective temperature of about 2*T*, which is analogous to that found in living matter^29,30^ and conducive to ring interlocking.^28^ Further details of the setup are given in sections S1 and S2 of the Supporting Information (SI).

The melt of unknotted and uncatenated rings was first equilibrated without activity, i.e., *T* = 1 for all monomers, see Figure 1a. At time *t* = 0, we turned on the activity for *m* rings and let the system evolve for durations much longer than the self-diffusion time of individual rings in a purely passive system, τ_diff_ = ⟨*R*_g_^2^⟩/6*D* ≃ 2.4 × 10^5^τ,^39^ see Figure 1a. The selected activity level induces simultaneous stretching and back folding of the rings, leading to various forms of entanglements^28^ that gradually immobilize the system. Figure 1b illustrates the resulting drop of cumulative mobility, μ(*t*). Here, μ(*t*) is defined as μ(*t*) ≡ μ(*t*, *t*_0_) = ⟨*g*_3_(*t*, *t*_0_)⟩/(*t* – *t*_0_), with *t*_0_ = 0 marking the onset of activity, and ⟨*g*_3_(*a*, *b*)⟩ representing the mean squared displacement between times *a* and *b* of the centers of mass of the rings. The average of *g*_3_(*a*, *b*) is taken over all rings, passive and active.

**Figure 1:**
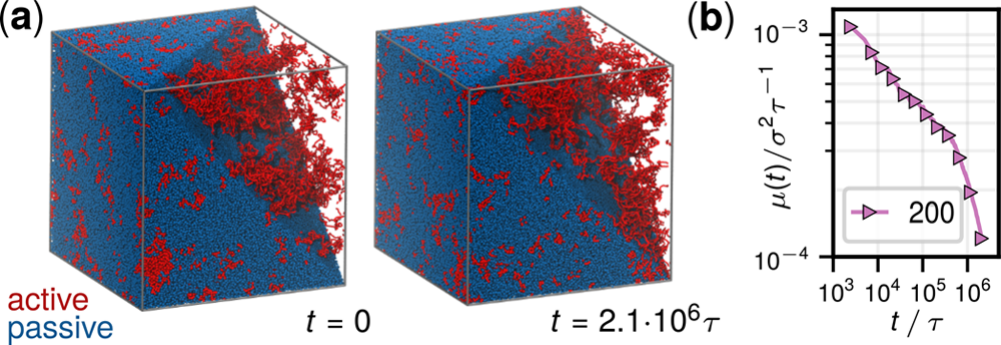
(a) Initial (equilibrated) and arrested states of a melt of *N* = 1600 rings, of which *m* = 200 are active. Red and blue colors are for active and passive rings, respectively. A portion of the periodic cubic simulation box is shown without the passive rings. (b) Time evolution of the ring mobility, μ(*t*), measured since the onset of activity for the case of *m* = 200.

To study the topological basis for the slowing collective dynamics, we developed a direct and general method for detecting deadlocked superstructures within a given ring melt configuration. The method, outlined in Figure 2, consists of the following steps, further detailed in SI, section S5. First, we examined all distinct ring pairs and excluded the linearly separable ones, evidently disentangled, from further consideration. We then examined a single retained pair at a time and identified all exposed three-monomer segments in each ring. The exposed state of a monomer was established from its linear separability from those of all other monomers. The separability test additionally yielded outward directions from the segment termini. We then used an iterative procedure to identify the two segments, one per ring, furthest from the center of mass of the ring pair and with divergent outward directions for all pairwise combinations of the four termini. Instances where no suitable termini could be identified were rare, typically amounting to fewer than five ring pairs in the entire melt. Next, removing the middle monomers of the segments turned each ring into an open chain with two exposed termini. Finally, the newly created termini of one chain were connected with those of the other using suitable arcs that extended the termini away from both rings (black arcs in Figure 2c). The reconnection of the two rings, which can be performed in two different ways, yields a single closed curve. The crucial observation is that, regardless of which reconnection method is chosen, the resulting curve is knotted if the original rings were deadlocked and unknotted otherwise, as sketched in Figure 2. Thus, detecting the knotted/unknotted state of the reconnected curve using topological invariants^40^ establishes the deadlocked/nondeadlocked state of the original rings.

**Figure 2:**
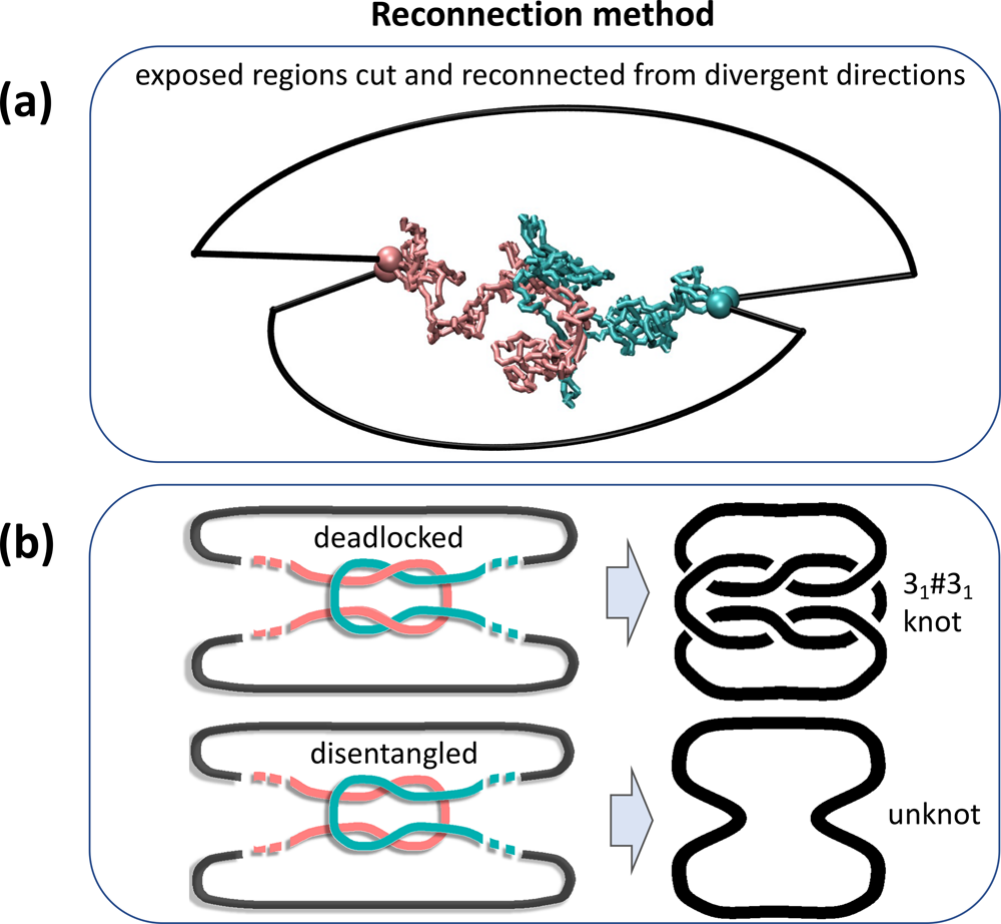
Reconnection method: “cross-wiring” two chains away from their overlapping regions returns a proper knot for deadlocked states and an unknot otherwise.

The reconnection method can be applied to ring pairs where meaningful reconnection points can be algorithmically identified away from the intermingling region, and thus is applicable only when the latter is sufficiently localized. It advances available alternative techniques,^41−44^ which primarily focus on detecting ring threadings, where one ring pierces the minimal surface of the other, but the converse is not necessarily true. The topological construction makes the reconnection method robust and different from the search of mutual ring threadings, a condition depending on geometrical details; see SI, sections S1 and S7. For the same reason, the method complements approaches more dependent on metric properties (see SI, section S3) and specific choices of points in space, such as primitive paths or persistent homology analysis.^2,17,43,45−47^ The method can be extended to systems with linear chains too by simply reconnecting their ends, making it possible to use bottom-up or top-down searches to pinpoint the deadlocked region. Finally, unlike indirect approaches,^48−50^ where interlockings are inferred from temporally persistent threadings^28^ (SI, section S7) or by comparing alternative trajectories,^51^ the reconnection works on instantaneous system configurations, enabling the tracking of deadlocks with arbitrary time resolution.

We used the reconnection method to probe the emergence of deadlocks in an evolving active system. The striking growth of interlockings is illustrated in Figure 3a, where the initial and the dynamically arrested states for *m* = 200 are compared. Pairwise deadlocks are schematically represented by segments joining the centers of mass of the rings. It is evident that the initially sparse interlockings eventually develop into an intricate and pervasive web of deadlocks, whose mechanism of formation is shown in Figures S1 and S2. The breadth of the deadlocks’ spectrum found in the melts is remarkable and vastly surpasses previously reported ones by variety and complexity,^24,28,52^ as illustrated in Figure 2b. The showcased examples were obtained by pulling interlocked rings in opposite outward directions with respect to the intermingling region, exposing the detected interlocking that would otherwise be elusive to visual inspection, see Figure S10.

**Figure 3:**
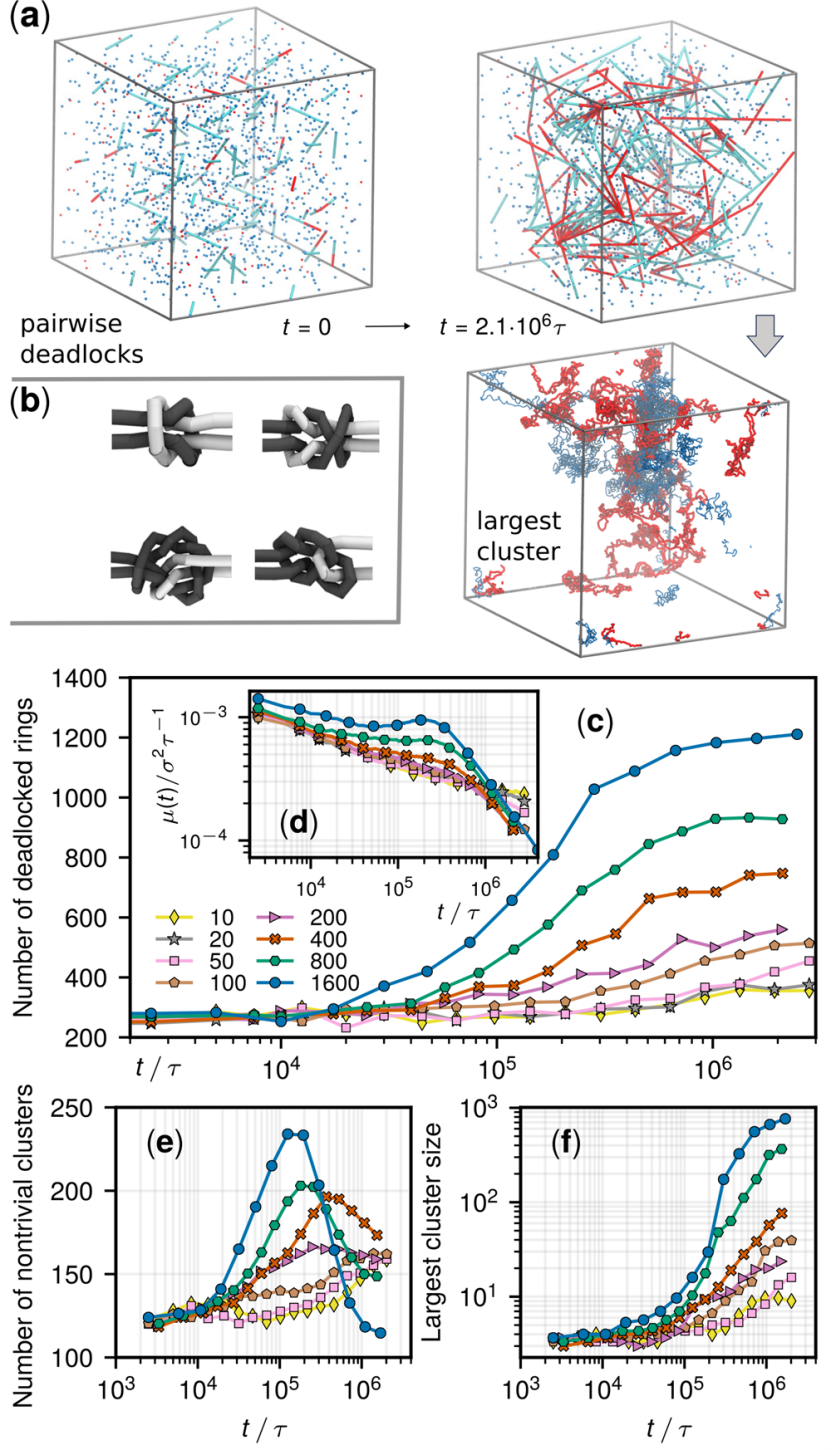
Simplified representation with only one bead per ring, with segments (colored according to the involved rings: red for active, blue for passive) joining deadlocked rings at equilibrium (*t* = 0) and at a later time. (b) Typical configurations of deadlocked regions obtained by pulling apart pairs of interlocked rings. Time evolutions of (c) the number of deadlocked rings, (d) the system’s mobility, (e) the number of nontrivial clusters of deadlocks, and (f) the size of the largest cluster of deadlocks. In all panels, different curves refer to systems with a different number *m* of active rings out of a total of *N* = 1600 rings.

The time evolution of the number of deadlocked rings is shown in Figure 3c. The curves in the semilog plot have a sigmoidal profile. For increasing activity level, *m*, the asymptotic number of interlockings grows, too, while the inflection point shifts to earlier times. For increasing *m*, the asymptotic values follow an approximate exponential saturation to the total number of rings in the melt, while the characteristic growth time follows a mild power-law decay with *m*, implying its nominal divergence for *m* → 0 (SI, section S6). The latter result is consistent with the expected absence of pervasive interlockings in purely passive ring melts.

For high levels of activity, *m* ≤ 200, the inflection points of the curves in Figure 3c occur for times comparable to the rings’ self-diffusion time, τ_diff_, and, additionally, approximately coincide with the major loss of mobility (Figure 3d). This indicates that rings only need to move by approximately their size to establish interlockings, leading to a significant loss of mobility. Indeed, even though the simulation time significantly exceeds τ_diff_, the totaled ring displacements since the onset of activity remains comparable to the ring size (SI, section S4).

A further noteworthy result from Figure 3d is that the most active systems become the least mobile in the long run, indicating that cooperative effects are involved in the growth and spreading of interlocking. We highlighted these mechanisms using a graph theoretical analysis to identify the clusters of deadlocked rings (SI, section S9). Figure 3e shows that the number of deadlocked clusters has an initial boost over times comparable to τ_diff_ and subsequent drops toward a steady-state value. This latter stage is more evident in systems with *m* ≥ 200, where interlockings develop faster. The drop is not caused by the system becoming disentangled, as the number of pairwise deadlocks would otherwise decrease. Instead, it originates from the coalescence of smaller clusters into larger ones. Indeed, the largest cluster can percolate through the entire system and grow to encompass a significant fraction of the rings, Figure 3a,f.

The progressive emergence of giant clusters is detailed in Figure 4, which shows the population of clusters of size *k* at different times for *m* = 800. The data reveal that disentangled rings (*k* = 1) can rapidly establish deadlocked pairs (*k* = 2), which reach their peak population at *t* ∼ τ_diff_, and then act as seeds for trimers and so on. The interconversions of the species occur even at late stages, i.e., for times much larger than τ_diff_, as reflected by the fluctuations of the population curves at all values of *m* (Figure S23). Despite this inherent stochasticity, the peak times of the various species, shown in Figure 4d, have the typical increasing trend of aggregation or assembly processes, where elementary binding events trigger a cascade of interconversions and the emergence of large and complex species.^53^

**Figure 4:**
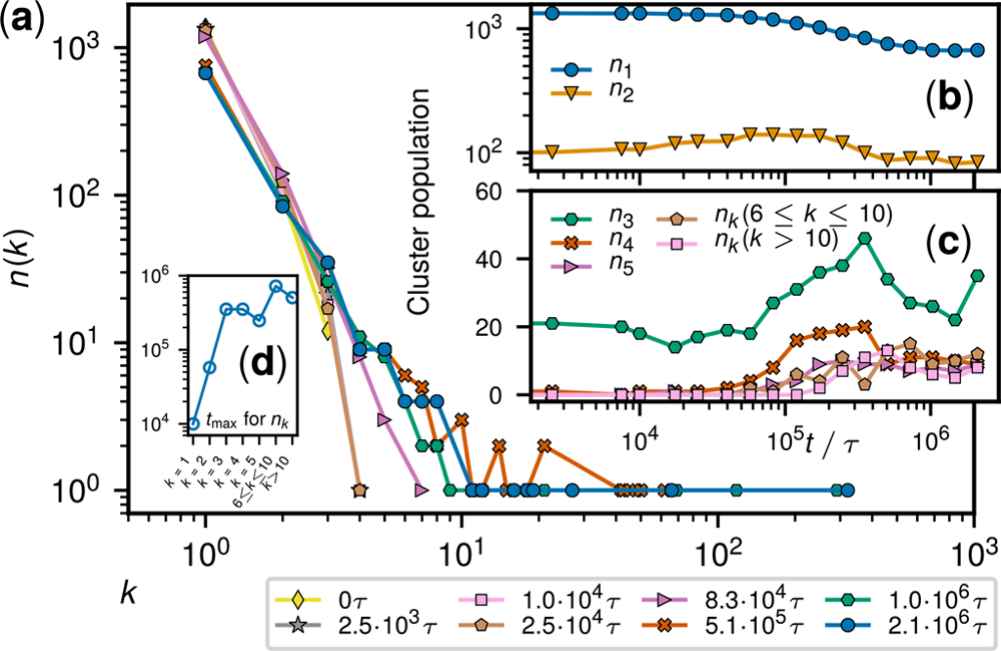
Evolution of deadlocked clusters for *m* = 800. (a) Log–log plot of *n_k_*, the number of clusters of *k* deadlocked rings at different times. (b, c) Time evolution of the populations of clusters of different sizes. (d) Times of the peak populations of clusters of different sizes, *t*_max_.

The cooperative interlockings emerging in highly active systems are particularly persistent (SI, section S8), making the formation of large clusters practically inevitable and irreversible. As a result, the Zipf-like population background is eventually offset by outlying giant clusters (Figure 4a). For instance, the rings in the largest cluster for *m* = 800 are initially 29 and become 321 at a late stage, a fifth of the entire system, see graph representations in Figure S22. Incidentally, we note that (i) the global arrested state emerges irrespective of boundary conditions,^54^ but the finite size of the simulation box, though significantly larger than any single ring, may limit the growth of clusters large enough to wrap across the periodic boundaries; and (ii) that clusters’ graphs can include cycles, and thus are not trees (Figure S22).

Figures 3 and 4 together indicate that the loss of ring mobility for large *m* occurs concomitantly with the increase of size and complexity of deadlocked clusters.

However, one intriguing aspect is that the loss of mobility is not limited to deadlocked rings but, strikingly, extends to nondeadlocked ones, too. At late times (*t*_*l*_ ≃ 1.5 × 10^6^τ) for *m* = 800, nondeadlocked rings are still numerous (∼650) and have mobility μ(*t*_*l*_, *t*_*l*_/3) ≃ 2.9 × 10^–5^σ^2^/τ, which is 10-fold smaller than in systems virtually free of deadlocks (2.5 × 10^–4^σ^2^/τ for *m* = 10) and hence indistinguishable from the mobility of deadlocked rings (SI, section S4 and Figure S8). The results establish that the intricate network of deadlocks can immobilize nondeadlocked rings, too.

To investigate how exactly this occurs, we extended the analysis to ring threadings, namely, instances where a ring’s minimal surface is pierced by one or more other rings.^28,54,55^ At the considered densities, threadings are pervasive even before the onset of activity, when each ring is threaded by 8 others on average (Figure S21). Notice that threaded and threading rings are not necessarily deadlocked and thus could move past each other.

The cooperativity of deadlocking and threading is detailed in Figure 5. After the onset of activity, the set of nondeadlocked rings is directly depleted by the expanding network of deadlocks, but is also increasingly threaded by it. The prominence of the latter effect is revealed by the noticeable growth of the number of nondeadlocked rings threaded by deadlocks, which proceeds until interlockings take over at *t* ∼ τ_diff_ = 2 × 10^5^τ. As a result, all nondeadlocked rings are threaded by deadlocked clusters at steady state.

**Figure 5:**
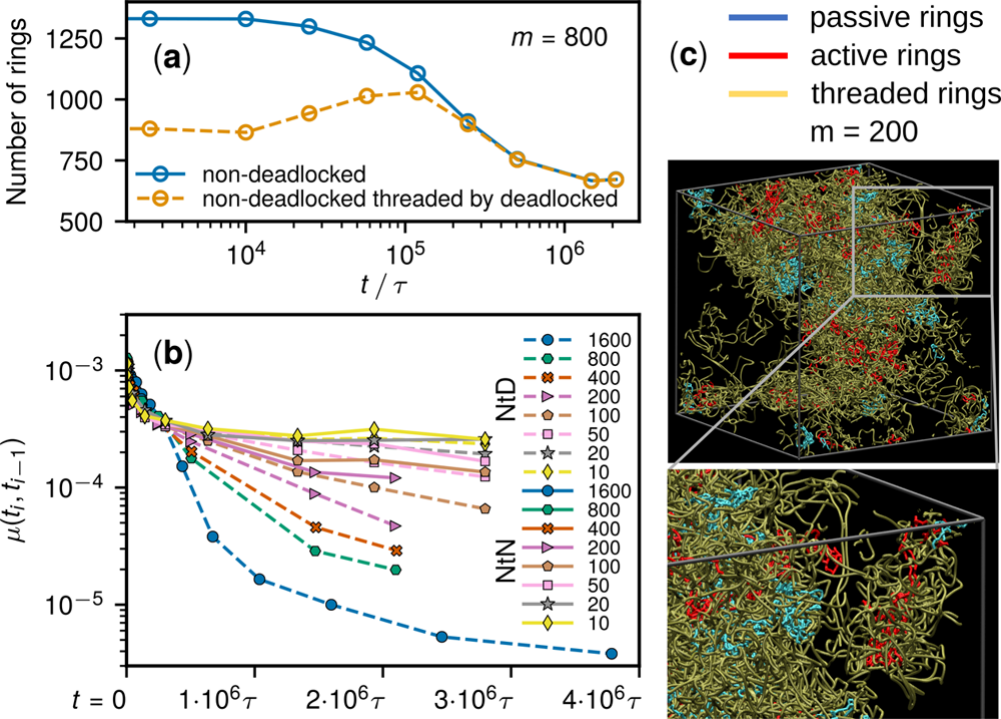
(a) Time evolution of the overall population of nondeadlocked rings (solid) and the subset threaded by deadlocks (dashed) for *m* = 800. (b) Time-dependent mobility μ(*t*_*i*_, *t*_*i*–1_), computed at log-spaced times *t*_*i*_ (symbols), of nondeadlocked rings that are threaded by deadlocked rings (“NtD”, dashed lines), and by nondeadlocked rings (“NtN”, solid lines) for varying *m*. In *m* ≥ 200 systems, the solid lines terminate earlier for the scarcity of the NtN population (<1% of the rings). (c) The same deadlocked cluster of Figure 1a, right (red, blue), is shown surrounded by the rings that it threads through (yellow). For clarity, the threaded rings have been smoothed with a shrinking elastic-band algorithm.^56^

The resulting entanglement is what causes the immobilization of nondeadlocked rings, as indicated by the increasing difference in mobility of nondeadlocked rings that are threaded or not threaded by deadlocked ones Figure 5b. A direct illustration is given in Figure 5c, which shows the largest cluster of Figure 1 (consisting of 34 rings) being entirely enveloped by the numerous (233) nondeadlocked rings that it threads through. The practical absence of mobile components makes it possible to consider the system as a genuine topological glass, distinguishing it from cluster glasses and conventional gels.^57,58^ Further, this establishes a causal link between the observed global dynamical arrest and the direct and indirect trappings of rings in the expanding network of deadlocks. The system is analogous to topological glasses with an artificially pinned fraction of rings,^59−61^ apart from the major distinction that here also the immobilization is self-organized, arises spontaneously by the deadlock entanglements.

Based on the above results, we can thus provide a topology-based account of the behavior of active ring melts. The onset of sufficient activity (*m* > 200) drives nearby rings to intermingle and then move away from one another in random directions, establishing numerous clusters of deadlocks, including giant ones, on time scales as small as the rings’ self-diffusion time. The formation of these clusters forces the codisplacement of the numerous deadlocked rings, directly hindering their motion and, indirectly, that of threaded rings, too, leading to the arrest of the entire system.

In further support of the above picture, we note that disconnecting the hubs of the deadlocked network by cutting open the rings with the highest degree of centrality does not reinstate the original mobility (SI, section S10). Thus, the loss of mobility is not simply due to the emergence of a single giant cluster that immobilizes a good fraction of the rings. This is consistent with the above notion that dynamical arrest is driven by the pervasive formation of numerous clusters of deadlocks dressed by threaded rings.

The reconnection method ought to be transferable to other stressed polymer systems, such as ring melts in shear, elongational, or more general flows, to elucidate the mechanistic underpinnings of time-dependent viscoelastic characteristics that are amenable to experiments. In particular, the presence of deadlocks in equilibrium that we discovered indicates their evolution must be markedly different in various nonequilibrium situations to allow for contrasting viscoelastic behavior in nonlinear flows, extensional thickening (due to the presence/formation of deadlocks) versus shear-thinning and seemingly no deadlocks in crazed ring glasses.^24,25,27^

A comparison with rheological simulations of ATG would give additional insights into the formation and evolution of deadlock entanglement types. Future applications include the investigation of viscoelasticity of macromolecules with more complex topologies, tadpoles, dumbells, knots, or single-chain nanoparticles,^21,62−64^ that could be prone to the formation of collective and cooperative forms of entanglement, including deadlocks and threadings which could be detected with the reconnection method. Further addressable systems would also be spatially confined or otherwise elongated chains,^65,66^ hydrogels,^67,68^ weaved materials,^69,70^ interphase chromosomes,^13,71,72^ and concentrated solution of DNA plasmids. The latter instance could also be used to validate our predictions for the effects of ring cuts, which could be introduced by topoisomerase enzymes.

## Supporting Information Available

Additional details about the model and system setup, the reconnection method, the clustering analysis, the dynamics of the system, and the evolution of deadlocks and threadings (PDF)
