# Supplementary material for: Topology-Based Detection and Tracking of Deadlocks Reveal Aging of Active Ring Melts
Source: ACS Macro Lett. 2024 Jan 10;13(2):124–9. doi: 10.1021/acsmacrolett.3c00567 (PMC10883035; doi:10.1021/acsmacrolett.3c00567)
Supplement: Supplementary file 1 — mz3c00567_si_001.pdf [file mz3c00567_si_001.pdf]

Supporting Information
Topology-based detection and tracking of deadlocks reveal aging of active ring melts

Cristian Micheletti,[1,*] Iurii Chubak,[2] Enzo Orlandini,[3] and Jan Smrek[4,†]

[1] *Scuola Internazionale Superiore di Studi Avanzati (SISSA), Via Bonomea 265, I-34136 Trieste, Italy*
[2] *Sorbonne Université CNRS, Physico-Chimie des électrolytes et Nanosystèmes Interfaciaux, F-75005 Paris, France*
[3] *Università degli studi di Padova, Dipartimento di Fisica "G. Galilei", Via Marzolo 8, I-35100 Padova, Italy*
[4] *Faculty of Physics, University of Vienna, Boltzmanngasse 5, A-1090 Vienna, Austria*

S1. MODEL DETAILS

We considered systems of $N = 1600$ active and passive ring polymers, each of $n_{\rm mon} = 400$ monomers (beads). The excluded volume interactions between monomers are treated via a truncated and shifted Lennard-Jones potential

$$U_{\rm LJ}(r) = \left(4\varepsilon\left[\left(\frac{\sigma}{r}\right)^{12} - \left(\frac{\sigma}{r}\right)^{6}\right] + \varepsilon\right)\theta(2^{1/6}\sigma - r), \tag{1}$$

where $\theta(x)$ is the Heaviside step function and σ the nominal monomer diameter. The polymer connectivity is provided by a standard finitely extensible nonlinear elastic (FENE) potential acting on consecutive monomers in a ring [1],

$$U_{\rm FENE}(r) = -\frac{1}{2}r_{\rm max}^2 K \log\left[1 - \left(\frac{r}{r_{\rm max}}\right)^2\right], \tag{2}$$

where $K = 30.0\varepsilon/\sigma^2$ and $r_{\rm max} = 1.5\sigma$, making the chains essentially uncrossable. In addition, triplets of consecutive monomers in a ring are subject to a bending-rigidity potential,

$$U_{\rm bend} = k_{\rm bend}(1 - \cos(\theta - \pi)), \tag{3}$$

where $k_{\rm bend} = 1.5\varepsilon$ and $\theta - \pi$ is the bending angle. These choices have been routinely used in many theoretical studies and applications [1, 2].

We studied systems where the N rings of $n_{\rm mon}$ monomers were packed at the total monomer density $\rho = 0.85\sigma^{-3}$. The number of active rings, m, varied from 10 to 1600.

All simulations were performed in the NVT ensemble using the large-scale atomic/molecular massively parallel simulator (LAMMPS) [3]. The Langevin equations of motion were integrated with a time step $\Delta t = 0.005\tau$, where $\tau = \sigma(M/\varepsilon)^{1/2}$, with different stochastic terms for passive and active rings [4]. For the passive monomers in active rings and all monomers in passive ones, the Langevin temperature was $T = 1$ (in units of ε, the Boltzmann constant is set to unity). The active rings contained a segment of 50 (consecutive) active monomers, corresponding to an eighth of the rings' contour length. To set the activity of these monomers, we fixed their Langevin temperature to $T_{\rm active} = 3$ (The coupling constants of both thermostats were $(2/3)\tau^{-1}$.). The choice $T_{\rm active} = 3$ is made based on two criteria (i) it maintains the stability of the simulation (no chain breaking or crossing) at a reasonably high Δt and (ii) the effective temperature of the system is about 2.0 (due to heat transfer between the cold and hot thermostats through the system) which agrees with the magnitude of the fluctuations of living chromatin observed in [5, 6]. The active segment promotes directional chain dynamics, where the rest of the chain is pulled behind. The specific dynamics allow for the formation of the deadlocks by the hot head wrapping around another ring(s), re-entering its own opening and pulling tight - see Fig. S1 for sketches of two of the possible formation mechanisms. The relation of the deadlocks to the threadings as defined by the minimal surface constructions [7] is explained in the corresponding caption. An actual example of deadlock formation from a simulated evolution of the melt with $m = 800$ is given in Fig. S2.

* cristian.micheletti@sissa.it
† jan.smrek@univie.ac.at

(a)

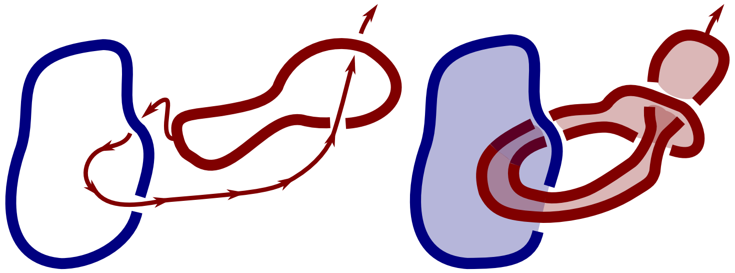

(b)

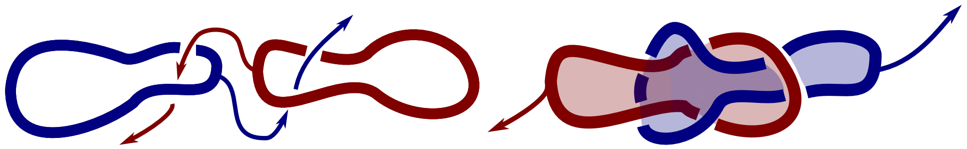

FIG. S1. Sketches of two of the possible deadlock formation mechanisms. In the deadlock conformations (right) the shaded regions represent the corresponding minimal surfaces. In the asymmetric case (a) the blue ring is threaded (its surface is pierced by the red ring), but the red ring is not threaded by the blue one (its surface is not pierced by the blue, but only by itself). In the symmetric case (b), the red and the blue rings are both, threading and threaded. The reconnection method we present in the main paper detects both cases.

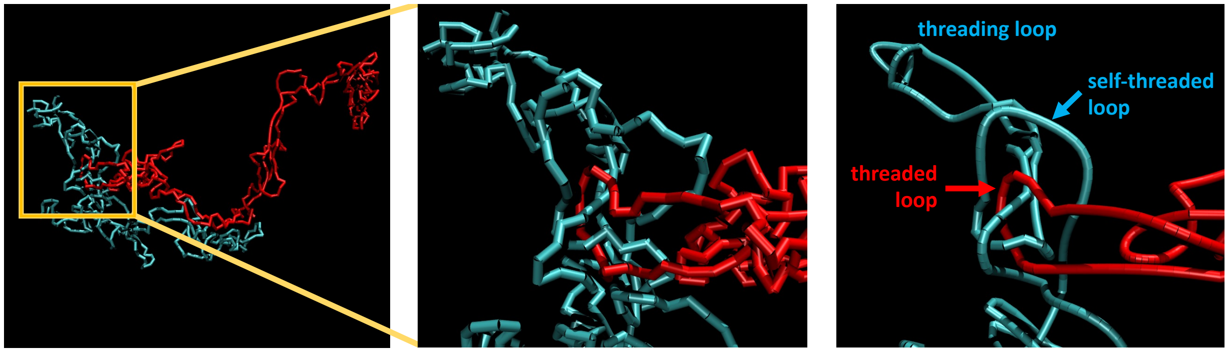

FIG. S2. Mechanism of deadlock formation. This example illustrates the typical mechanism of deadlock formation, where a (threading) loop passes through a loop of the partner ring and then through a further loop of its own ring. The middle panel provides an enlargement of the deadlocked region, and the rightmost panel is a smoothed version of the same configuration. The example is from the $m = 800$ case at $t = 3.5\,10^5\tau$. Both rings are active, and the tip of the threading loop is part of the active region of its ring.

S2. INITIALIZATION

We initialized the system of active and passive rings in a state obtained from an equilibrated melt exclusively made of passive rings. To obtain the equilibrated melt, we first created a dilute version of the system of rings. For this step, we used a simple planar geometry for the rings and placed them in the box in a non-overlapping and randomly oriented manner. The system was then compressed over a relatively short timespan $t_{\text{compression}} = 500\tau$ by rescaling distances at a constant rate (once per τ), until reaching the desired density $0.85\sigma^{-3}$. The preservation of the non-concatenated state of the system was verified by computing the linking number of all pairs of proximal rings after compression. We then relaxed the system of all passive rings with NVT simulations of duration $t_{\text{eq}} = 5.10^5\tau \simeq 2\tau_{\text{diff}}$, which suffices to reach equilibrium and the diffusive regime for the considered chain lengths [8]. For computational efficiency, the procedure was used to generate an equilibrated melt of 200 passive rings, which was then duplicated along each of the three Cartesian axes to generate the initial configuration of 1600 rings.

S3. CONFORMATIONAL PROPERTIES

Details of the rings' conformational properties are presented in Figures S3, S4, S5. The plots illustrate the dependence of the gyration radius R_{g} and the (adimensional) asphericity parameter a on the number of active rings, m.

The two observables, R_{g} and a, were computed from the three ranked eigenvalues λ_i ($\lambda_1 \geq \lambda_2 \geq \lambda_3$) of the ring's gyration tensor, $G_{\alpha\beta} = \frac{1}{n_{\text{mon}}}\sum_{i=1}^{n_{\text{mon}}}(r_\alpha^{(i)} - R_\alpha)(r_\beta^{(i)} - R_\beta)$ with $r_\alpha^{(i)}$ is the α-th Cartesian component of the position vector of the ith monomer, $\boldsymbol{r}^{(i)}$, and $\boldsymbol{R}$ is the center of mass position of the ring,

$$R_{\text{g}} = \langle \lambda_1 + \lambda_2 + \lambda_3 \rangle^{1/2}\,, \tag{4}$$

$$a = \left\langle \frac{\lambda_1 - \frac{1}{2}(\lambda_2 + \lambda_3)}{\lambda_1 + \lambda_2 + \lambda_3} \right\rangle, \tag{5}$$

where the brackets $\langle \cdots \rangle$ denote ensemble averaging. The shape and size parameters were averaged for $t > 1.8{\cdot}10^6\tau$, when most activity-induced deformations had set in. In addition, we define the instantaneous value of the radius of gyration $\hat{R}_{\text{g}} = (\lambda_1 + \lambda_2 + \lambda_3)^{1/2}$.

As shown by the plots, activity affects the shape and size of both active and passive rings. Fig. S3 shows the gyration radius of active and passive rings, distinguishing whether they are involved in deadlocked clusters or not. R_{g} of active rings is about twice as large in comparison to that of rings in an equivalent equilibrium system (Fig. S3(a)-(c)). By contrast, the R_{g} of passive rings is only moderately larger (by about 25% for $m = 800$). Furthermore, the $\hat{R}_{\text{g}}$ probability distribution differs considerably for active and passive rings; a fact reflected by the bimodal cumulative $\hat{R}_{\text{g}}$ distribution (Fig. S4). Active rings take on highly aspherical, elongated conformations, as revealed by their larger a-values compared to passive rings (Fig. S5(a)-(c)). Compared to equilibrium, the asphericity of passive rings is enhanced by about 15% (Fig. S5(a)-(c)).

Comparison of R_{g} and a in Fig. S3(e)-(f) and Fig. S5(e)-(f) reveals that belonging or not to a deadlocked cluster does not significantly affect active/passive rings' size and shape. The R_{g} averaged over all rings in a deadlocked cluster is larger compared to that of rings not in a cluster (Fig. S5(d)), indicating that active rings (larger than passive ones) are more likely to be involved in deadlocks. The same effect is observed in the radius of gyration distributions in Fig. S4. Furthermore, we find that rings with the highest degree centrality in a deadlocked cluster have a flatter $\hat{R}_{\text{g}}$ distribution (see red lines in Fig. S4). All these observations suggest that conventional metric observables are not viable indicators of whether a ring is involved in a deadlocked cluster.

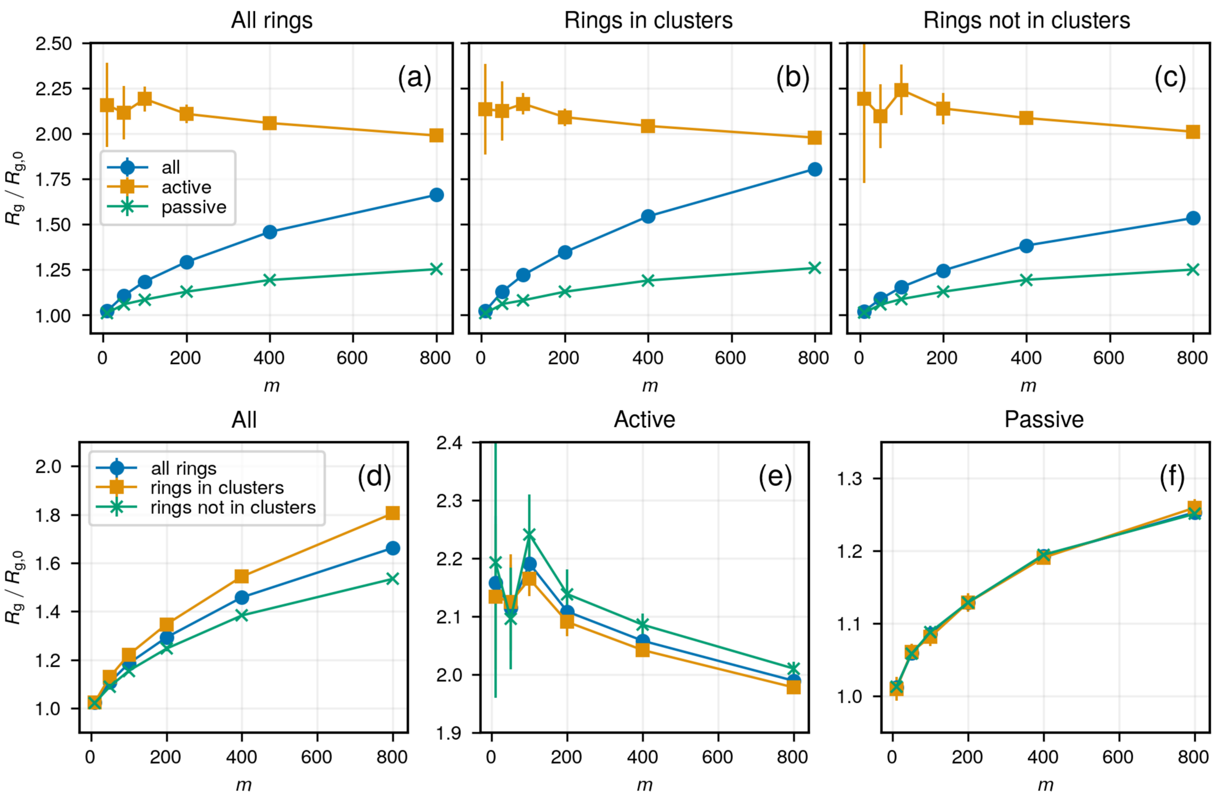

FIG. S3. Mean radius of gyration R_g as a function of m for (a) all rings, (b) rings belonging to deadlocked clusters, (c) rings not in deadlocked clusters. In (a), (b), (c), we show separately R_g for active (yellow squares) and passive (green crosses) rings, as well as the ensemble-averaged value (blue circles). R_g as a function of m for (d) all rings in the system, the subsystem of active (e) and passive (f) rings. In (d), (e), (f) we show separately R_g for deadlocked (yellow squares) and not deadlocked (green crosses) rings, as well as the ensemble-averaged value (blue circles). R_g is normalized by the equilibrium value of the radius of gyration $R_{\mathrm{g},0} = 7.27\sigma$ [9].

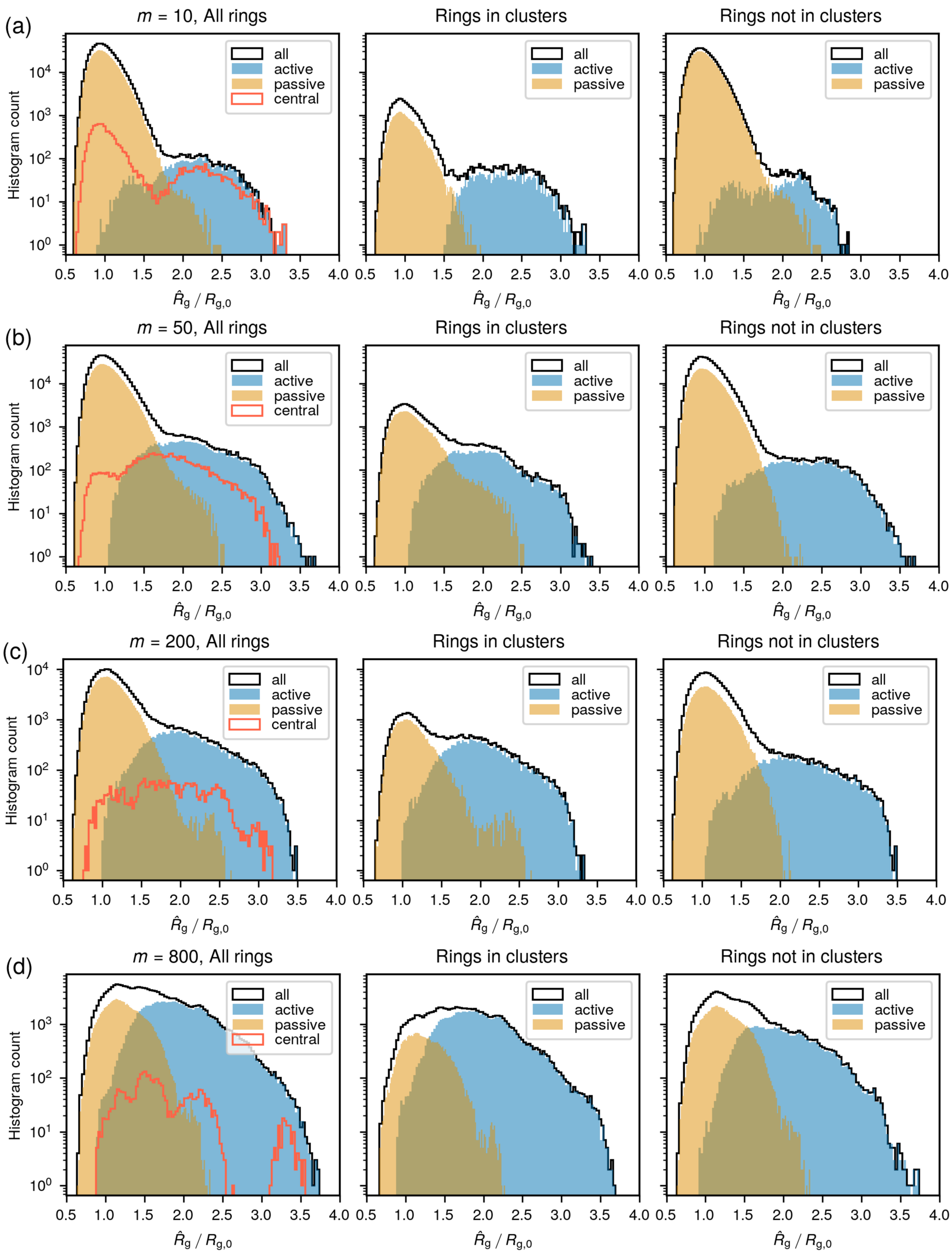

FIG. S4. Distribution of the instantaneous radius of gyration $\hat{R}_g$ for systems with $m = 10$ (a), 50 (b), 200 (c), and 800 (d) partly active rings. The system contains 1600 rings, each having 400 beads. The distributions in the left, central, and right columns correspond to all rings in the system, those that belong to deadlocked clusters, and those that do not belong to the clusters, respectively. The yellow areas indicate completely passive rings, whereas the blue ones indicate active rings. The red curves indicate the distribution of $\hat{R}_g$ for a subset of the most central rings in deadlock clusters. The x-axes are normalized by the equilibrium value of the mean radius of gyration $R_{g,0} = 7.27\sigma$ [9].

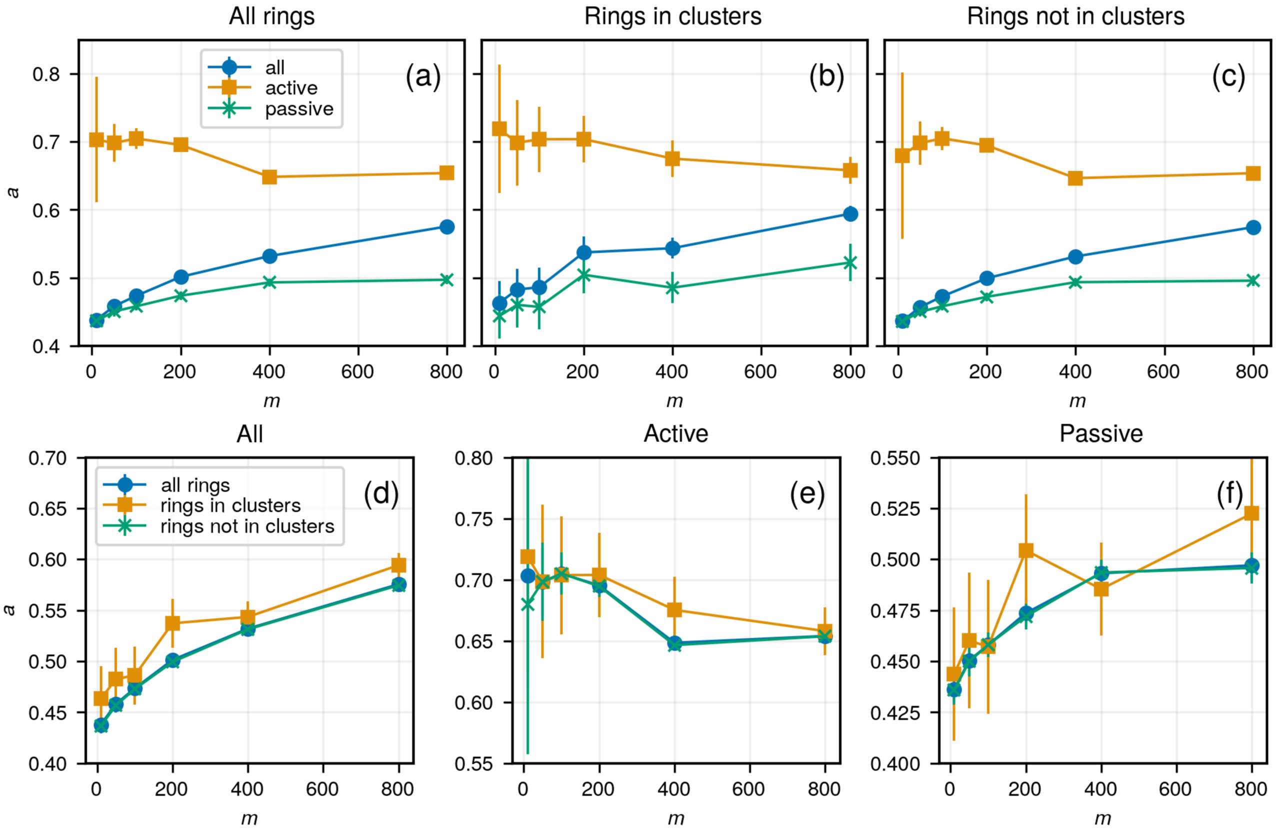

FIG. S5. Asphericity parameter a as a function of m for (a) all rings, (b) rings belonging to deadlocked clusters, (c) rings not in deadlocked clusters. In (a), (b), (c) we show separately a for active (yellow squares) and passive (green crosses) rings, as well as the ensemble-averaged value (blue circles). a as a function of m for (d) all rings in the system, the subsystem of active (e) and passive (f) rings. In (d), (e), (f) we show separately a for deadlocked (yellow squares) and not deadlocked (green crosses) rings, as well as the ensemble-averaged value (blue circles).

S4. DYNAMICAL PROPERTIES

We assessed the system's dynamics using the ensemble-averaged mean-square displacements (MSD) of the rings' centers of mass (CoM), $g_3(t)$:

$$g_3(t;t_0) = \frac{1}{N}\sum_{i=1}^{N}\left[\boldsymbol{R}_i(t+t_0) - \boldsymbol{R}_i(t_0)\right]^2, \tag{6}$$

where t_0 is the considered time origins and $\boldsymbol{R}_i(t)$ is the CoM position of the i-th ring at time t measured relatively to the CoM of the whole system at that time.

After large rearrangements occurred (before $t_0 = 10^6\tau$), we additionally perform time-averaging of the MSDs, yielding $\langle g_3(t)\rangle$:

$$\langle g_3(t;t_0)\rangle = \frac{1}{T-t_0}\int_{t_0}^{T} g_3(t;t')\,\mathrm{d}t', \tag{7}$$

The activity-induced vitrification of the system is manifested by the significant decrease of $\langle g_3\rangle$ for an increasing number of active rings, m, see Fig. S6(a).

Only for sufficiently few active rings, $m \leq 50$, $\langle g_3\rangle$ shows an approximate diffusive regime over the considered time scales. Rings in deadlocked clusters are systematically less mobile than the average (Figs. S6 and S8) due to the various and stringent topological constraints to which they are subject. A notable and perhaps counterintuitive effect is that active rings can even be less mobile than passive ones precisely due to their higher likelihood to participate in numerous concurrent deadlocks, see Fig. S7.

As we see from the $\langle g_3\rangle$ data, the systems evolve even after t_0 although very slowly (estimated relaxation time is six orders of magnitude larger than that of equilibrium [4]). As shown in the main text, this quasi-steady state is aging to become more deadlocked and entangled. As all the rings are non-concatenated, it is, in principle, possible that a deadlock would be undone. Such a process is likely to be exponentially suppressed as it would require the active segment to trace back its passive tail to avoid forming a deadlock on the way.

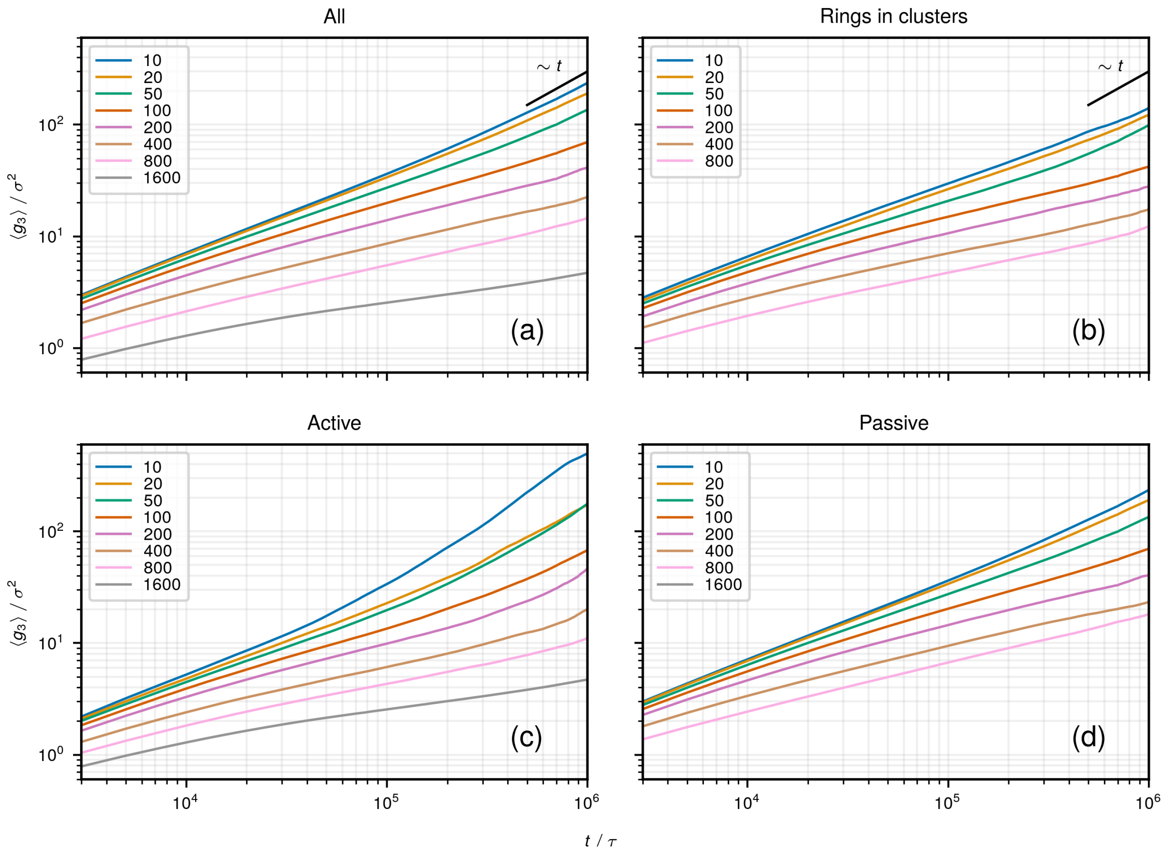

FIG. S6. Late-stage mean-square displacements in systems with a different number of active rings m (indicated in the legend) averaged over for (a) all rings in the system, (b) rings participating in deadlocked clusters, (c) only active rings, (d) only passive rings.

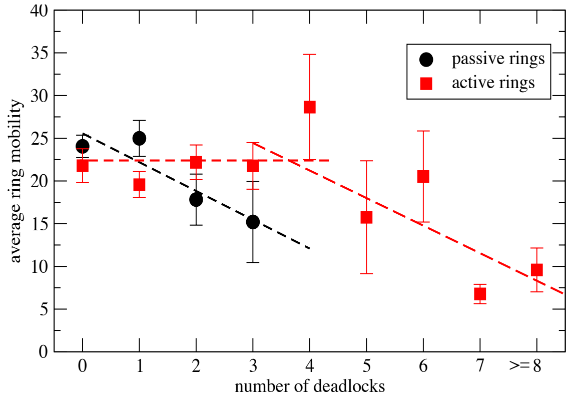

FIG. S7. Scatter plot of the average mobility of active and passive rings with a varying number of deadlocks for a system with $m = 800$ active rings. The dashed lines are guides to the eye.

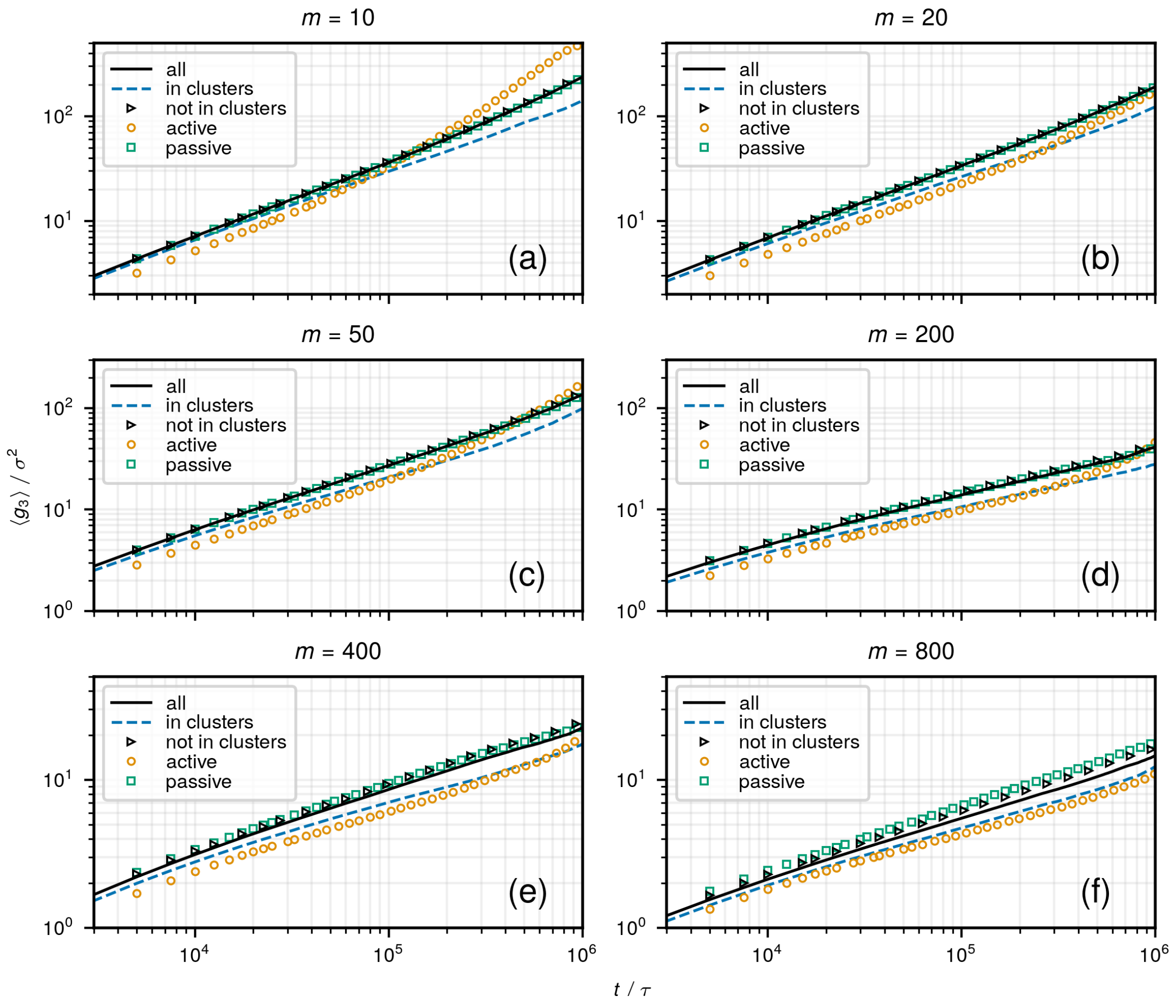

FIG. S8. Late-stage mean-square displacements in systems with a different number of active rings m = 10 (a), 20 (b), 50 (c), 200 (d), 400 (e), 800 (f) averaged over all rings in the system (black solid line), rings participating in deadlocked clusters (dashed blue line), rings not in deadlocked clusters (black triangles), only active rings (yellow circles), only passive rings (green squares).

S5. RECONNECTION METHOD

Pairs of deadlocked rings were singled out with the reconnection method outlined in the main text. The method consists of cutting open the rings away from their region where they intermingle and then cross-wiring the resulting open curves by bridging the newly created termini of one chain with those of the other. The reconnection procedure returns a single closed curve, which is knotted if the original rings were deadlocked and unknotted otherwise, see Fig. S9.

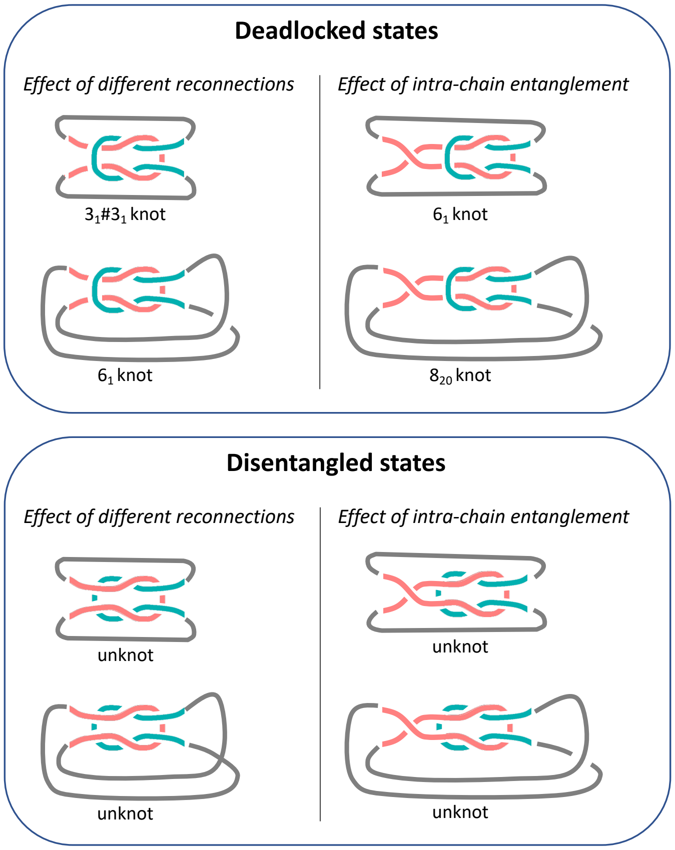

FIG. S9. Effect of different reconnection choices and intra-chain entanglement on the topology of the resulting reconnected ring. Deadlocked states yield knotted reconnected rings, though the (non-trivial) topology can vary with how the termini are reconnected and with intra-chain entanglement. Disentangled states instead yield unknotted reconnected rings.

Note that the key property to consider is the unknotted versus the knotted state of the reconnected curve, as the topological complexity of the knot does not necessarily correlate with the complexity of the deadlock. In fact, different knot topologies [10] - though always non-trivial - can be obtained with alternative choices for reconnecting the chains' termini, see the top left panel in Fig. S9. In addition, as illustrated in the top right panel of Fig. S9, intra-ring entanglement can also add to the topological complexity of the reconnected curve in the case of deadlocks. For non-deadlocks, instead, the unknotted topology of the reconnected curve is not affected by alternative reconnection choices and intra-chain entanglement, see bottom panels of Fig. S9.

To efficiently implement the deadlock profiling procedure, we first listed all distinct pairs of rings in a given snapshot of the system. We then eliminated the pairs that were manifestly disentangled. This step was performed by checking the linear separability of any two rings, that is, whether at least one plane existed that subdivided the space into two regions, each occupied by only one of the two rings.

We next processed each of the remaining ring pairs in turn. We identified all exposed segments of three monomers for each ring in the pair. Below we shall indicate with $\vec{r}_i^A$, $\vec{r}_{i+1}^A$ and $\vec{r}_{i+2}^A$ and $\vec{r}_j^B$, $\vec{r}_{j+1}^B$ and $\vec{r}_{j+2}^B$ the coordinates of the exposed triplets of monomers in ring A and B. The exposed condition for a segment was tested by assessing the linear separability of each of the three monomers from all other beads of *both* rings. The normal to the separating plane (which is not unique) identifies the local outward directions, $\vec{w}_i^A$, $\vec{w}_{i+1}^A$ etc. along which one can move without intersecting the convex hull of both rings.

We then proceeded iteratively to identify the two segments, one per ring, where the rings can be cut and prolonged outward along diverging, i.e. non-interfering, directions. To do so, we first picked for each ring the exposed triplet furthest from the center of mass of the entire two-ring system (Fig. S10a) . Next, we tested that the outward-directed vectors of all four termini are divergent with respect to one another (Fig. S10b). In case the mutual divergence test was not passed, the segment pair was marked as nonviable, and the search was repeated. Very few (< 5) iterations of the search procedure typically sufficed to identify a solution. The cases where no suitable closure was found were exceptional, typically amounting to fewer than 5 ring pairs in vitrified snapshots; such pairs were not included in the analysis.

Next, we removed the middle monomers $i+1$ and $j+1$, thus turning each ring into an open chain with two exposed termini. The newly created termini of one chain were then paired with those of the other and connected with a suitable arc. For each connected pair, the arcs were constructed by prolonging the termini along their outward directions up to the intersection with a sufficiently large sphere centered on the center of mass of the two-chain system. Two different radii were used for the termini pairs. The two intersection points were then connected with an arc on the sphere (Fig. S10c).

Finally, the knotted state of the resulting reconnected curve was established by evaluating the Alexander polynomial, $A(t)$, in $t = -1$ and $t = -2$ [10]. Typical configurations of deadlocked rings detected by the reconnection method are shown in Fig. S10d.

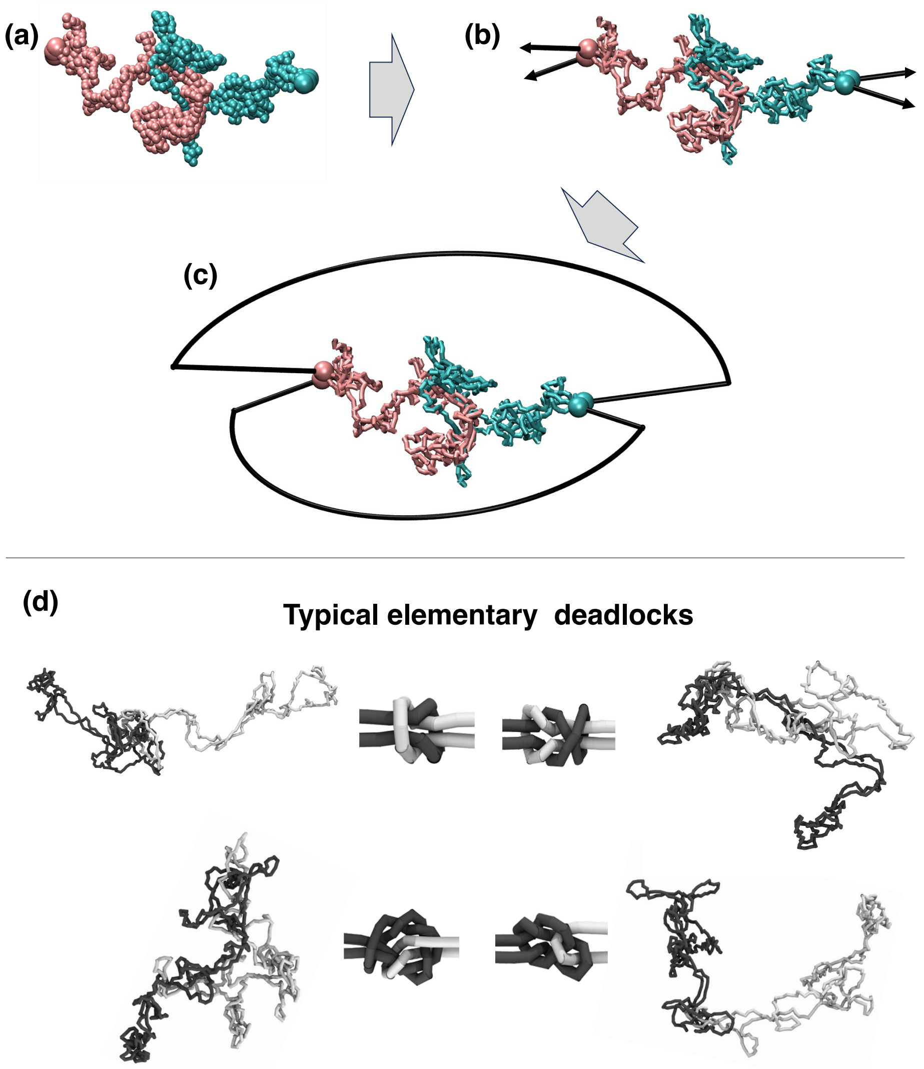

FIG. S10. (a-c) Schematic representation of the main steps of the reconnection procedure, see text. (d)Typical configurations of deadlocked rings detected by the reconnection method. Pulling the rings apart yields the tight interlockings at the center of the figure, confirming and exposing the detected interlockings.

S6. SCALING PROPERTIES OF THE EVOLUTION OF DEADLOCKS

Fig. S11 presents the evolution of the number of pairwise deadlocks in ring melts at different activity levels. The plot complements Fig. 3c of the main text, which shows the time evolution of the number of deadlocked rings. Notice that because a ring can be deadlocked with multiple other rings, the number of deadlocked rings is typically smaller than twice the number of pairwise deadlocks.

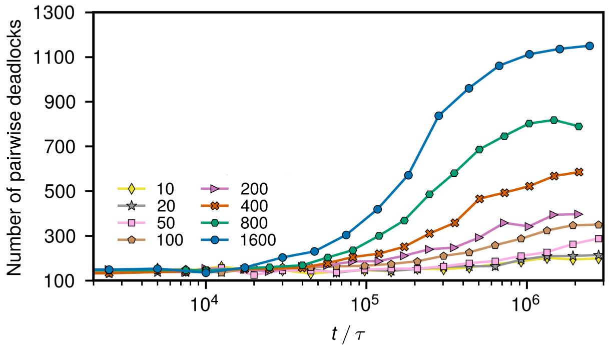

FIG. S11. Time evolution of the number of pairwise deadlocks for systems with varying number of active rings, m. Time is measured since the activity onset.

To characterize how the abundance of deadlocks grows both with time and with the number of active rings, m, we carried out a scaling analysis of the number of non-deadlocked rings, which decays with time.

The time evolution of the number of non-deadlocked rings for all considered activity levels is shown by the data points (filled symbols) in Fig. S12.

The continuous lines are best fits to the data with an exponential decay whose functional dependence on time, t, is:

$$f(t) = n_a + (1366 - n_a)\exp(-t/t_0) \ , \tag{8}$$

where the two fitting parameters are t_0, the characteristic decay time, and n_a, the limiting number of deadlocks for asymptotically-large times. The expression of the best fit is constrained to the initial number of non-deadlocked rings (1366) for $t = 0$.

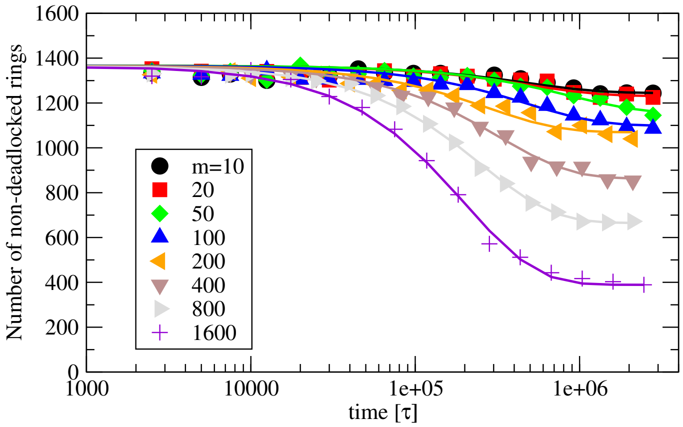

FIG. S12. The data points refer to the time evolution of the number of non-deadlocked rings for systems with different numbers of active rings. The continuous lines are best fit to the data based on an exponential decay to a limiting value, see text.

The m-dependent scaling properties of the asymptotic value, n_a, and the characteristic decay time, t_0, are shown in Fig. S13. Best fits to the data (solid lines), indicate that, for the considered system size and range of m, n_a follows an approximate exponential decay with m, while t_0 follows a mild power-law decay. The latter property, which indicates a nominal divergence of t_0 for $m \to 0$, is qualitatively consistent with the expected absence of percolating clusters of deadlocked rings in passive ring melts. For very large systems with high fractions of active rings, it appears plausible that the observed scaling could cross over to a plateau defined by a suitable microscopic characteristic time.

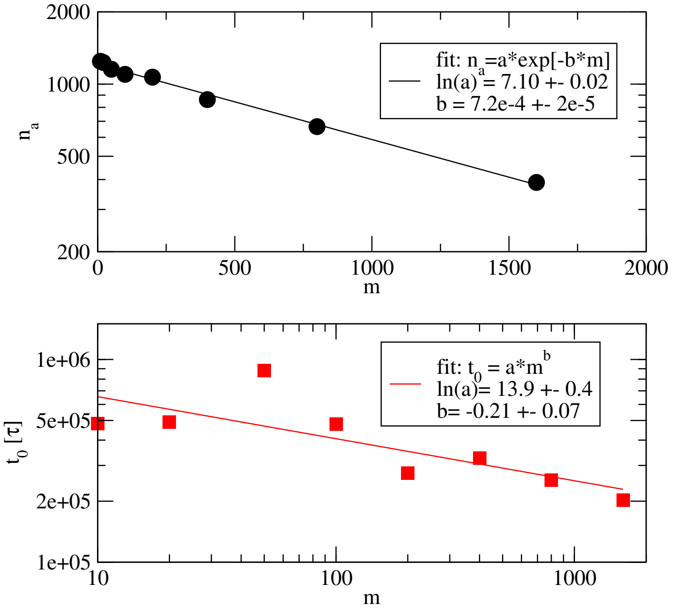

FIG. S13. The data points refer to the dependence of the number of non-deadlocked rings on the number m of active rings in the system. The continuous lines are best fit to the data based on an exponential decay to a limiting value, see text.

S7. PERSISTENT THREADINGS

In this section, we discuss the analysis of ATG entanglements by comparing the reconnection method and the persistent threadings method of ref. [4]. First, as is clear from the description of the method in sec. S5 and the sketches in Figs. S1 and S9 the deadlocks are not equivalent to mutually threaded rings even if persistent. Next, we recall that the reconnection method detects a deadlock from an instantaneous configuration, while to detect if certain threadings are persistent, one has to exploit the dynamical evolution of a system. Specifically, the latter method stems from the consideration [4] that, once established, crucial forms of entanglements associated with ATG vitrification can be extraordinarily long-lived and accompany the system evolution almost indefinitely. Building on this notion, the persistent threading method infers the presence of crucial entanglements *via* long-lived instances where one ring latches onto the contour of another one [4, 11]. Therefore, it is interesting to establish a connection between the two methods, e.g., to ascertain whether the deadlocks instantaneously identified with the reconnection method are persistent or establish which fraction of persistent threadings correspond to deadlocks.

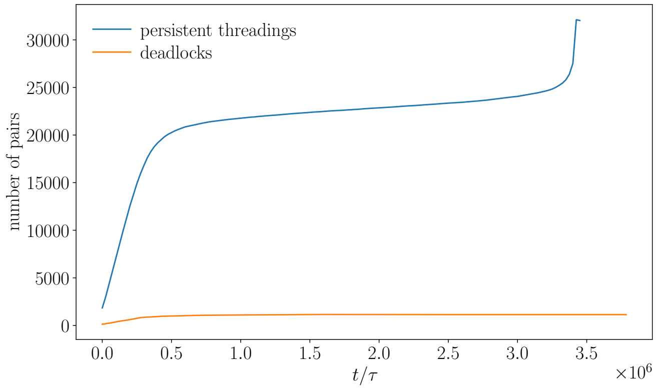

FIG. S14. Comparison of the number of pairs in the set of persistently threading and deadlock sets as a function of time.

To this end, we considered a fully active system ($m = N = 1600$) and analyzed the threadings of 139 snapshots distributed along the entire trajectory of duration $3.5.10^6\tau$. For each snapshot, we determined the persistent threadings, i.e. those lasting until the end of the trajectory.

The time evolution of the number of ring pairs in the persistent threadings is shown in Fig. S14 and features two rapid growths. The first one is at early times when the system is rapidly changing and forming threadings in response to the onset of activity. The second one is at late times and is artificial, resulting from temporary threadings being nominally counted as persistent merely due to the short remaining time to the end of the trajectory. The number of persistent threadings is relatively stable between these two regions. The corresponding depth of the threadings is also stable after the initial $2.10^5\tau$. We ascertained this by considering the so-called separation length of the threading ring $L_{\rm sep}$, a measure of threading depth, and computing the t-test on the samples at different times. The probability of rejecting the null hypothesis that the samples originate from distributions with the same mean is $p < 10^{-6}$. The $L_{\rm sep}$ is computed with the aid of the minimal surfaces on the ring. We counted a piercing of one surface by another ring as threading. Subsequent n piercings of the minimal surface divide the threading ring into so-called threading sections of lengths $L_{\rm th_i}$ for $i = 1, 2, ..., n$, where n is even, because the rings are unlinked (every piercing has to have a partner that pierces the surface in the opposite direction otherwise the rings would be linked). The lengths of the threading sections are obtained as the segment lengths between subsequent surface piercings. Based on $L_{\rm th_i}$ we can define the separation length as $L_{\rm sep} = \min(\sum_{i\in{\rm even}}^{n} L_{\rm th_i}; \sum_{j\in{\rm odd}}^{n} L_{\rm th_j})$, which characterizes how much of the threading ring resides on one side of the threaded ring's surface - see sketch in Fig. S15. More details of the threading method are presented in [4, 7, 11].

The $L_{\rm sep}$ distribution for persistent and temporary threadings is presented in Fig. S16 at time $2.45\,10^6\tau$. Shallow threadings (small $L_{\rm sep}$) dominate the set of temporary threadings, while persistent ones have practically flat distributions.

We next directly compared the sets of persistent threadings and deadlocks at time $2.45\,10^6\tau$ and found that the deadlocks *are a stringent subset of the persistent threadings*, with the only exception of 3 deadlock pairs, out of about 1100, that eventually unthread over time. Remarkably, the $L_{\rm sep}$ distribution computed for deadlocks at time $2.45\,10^6\tau$ is almost superposed to that of persistent threadings, too, see S16.

To further investigate the temporal relationship between deadlocks and persistent threadings, we computed the

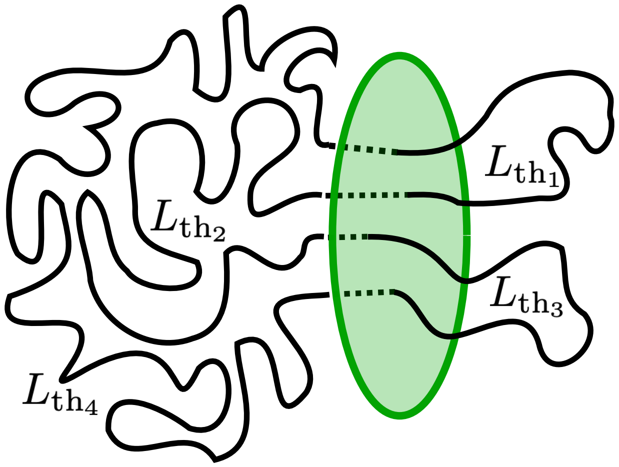

FIG. S15. Sketch of threading rings. The green ring's minimal surface is pierced by the black ring four times, which splits the threading ring into segments L_{th_1} through L_{th_4}. The separation length L_{sep} (see the formal definition in section S7) in this case would be the sum $L_{\mathrm{th}_1} + L_{\mathrm{th}_3}$, because the total length is smaller than the $L_{\mathrm{th}_2} + L_{\mathrm{th}_4}$. The L_{sep} therefore reflects the measure of the asymmetry of the distribution of the total length of the threading ring's segments on the two sides of the minimal surface of the threaded ring.

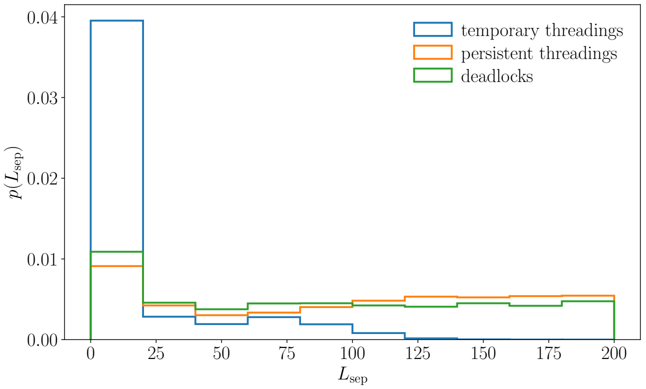

FIG. S16. Distribution of the separation length L_{sep}.

number of deadlocks at time t that are also in the set of persistent threadings at a given time $\bar{t}$, $n_d(t|\bar{t})$. The time evolution of this quantity for $\bar{t} = 2.45 \times 10^6$ is shown in Fig. S17, where it is normalized to the instantaneous overall number of deadlocks, $n_d(\bar{t})$. Even at early times, the large majority ($> 87\%$) of deadlocks - which are few in absolute terms - belong to the set of persistent threadings. The result suggests that the few deadlocks in the initial equilibrium state are seeds to what eventually becomes the vitrified entangled state.

Fig. S18 shows the fraction of deadlocks that persist (as deadlocks) as time progresses (notice the logarithmic temporal spacing). At early times, about 30% of the deadlocks vary between two consecutive time milestones, though these variations are primarily due to transient deadlocks being traded for persistent ones (see Fig. S17). Again, this result supports the persistence or irreversibility of deadlocks beyond $t \simeq \tau_{\mathrm{diff}} = 2.4 \times 10^5 \tau$.

Finally, we looked at the number of deadlocks at time t that do not belong to the set of persistent threadings at that same time t, $n_d^{\neg PT}(t)$. In Fig. S19, we plot the ratio $n_d^{\neg PT}(t)/n_d(t)$ as a function of time, showing that the deadlocks form a substantial fraction of the set of persistent threadings at any time, even at equilibrium, where they constitute 89% of the total. This further supports the view that deadlocks in equilibrium form the seed of the vitrified cluster.

To summarize, the deadlocks are a stable and stringent subset of persistent threadings. This result adds *a posteriori* notable temporal connotation to the interlockings detected with the reconnection method, a property that might be exploited in future studies.

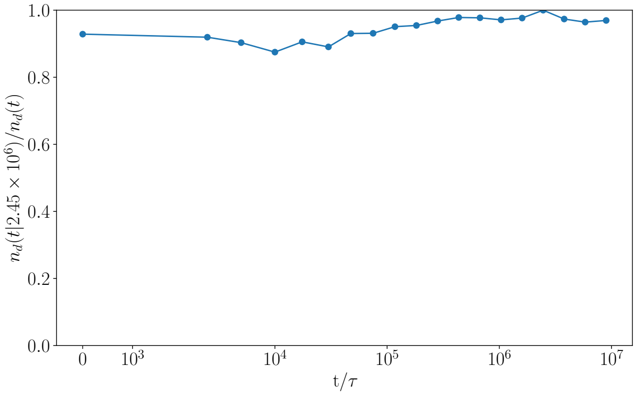

FIG. S17. Fraction of deadlocks at time t that is in the set of persistent threadings at time 2.45×10^6. The time scale is linear for $t \leq 2500\tau$ and logarithmic otherwise.

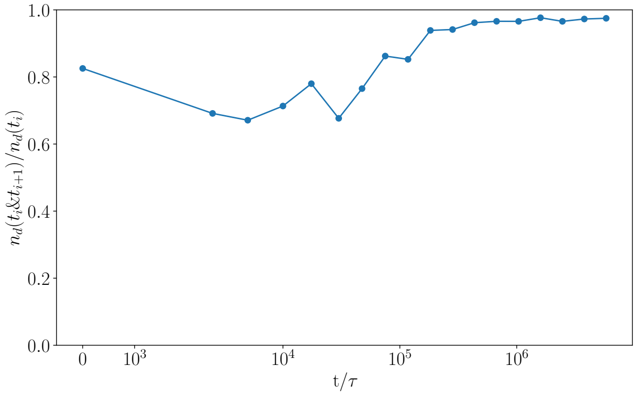

FIG. S18. Fraction of deadlocks in a snapshot at time t_i that is also in the set of deadlocks at the next snapshot t_{i+1}. the snapshots are distributed logarithmically in time. The time scale is linear for $t \leq 2500\tau$ and logarithmic otherwise.

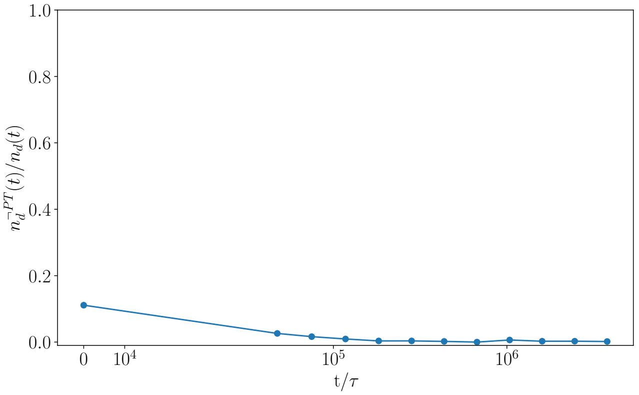

FIG. S19. Fraction of deadlocks at time t that do not belong to the set of persistent threadings at that time. The time scale is linear for $t \leq 47000\tau$ and logarithmic otherwise.

S8. PERSISTENCE OF DEADLOCKS

Deadlocked states can fully or partially unravel when one or more of the involved rings disengage from the interlocked clusters. Disengagements are frequent in the initial stage of the dynamics. However, they become rarer as time progresses when rings are simultaneously interlocked with multiple other members of their cluster. This is illustrated in Fig. S20, which shows the dropping rate at which individual rings go from a deadlocked to a free state.

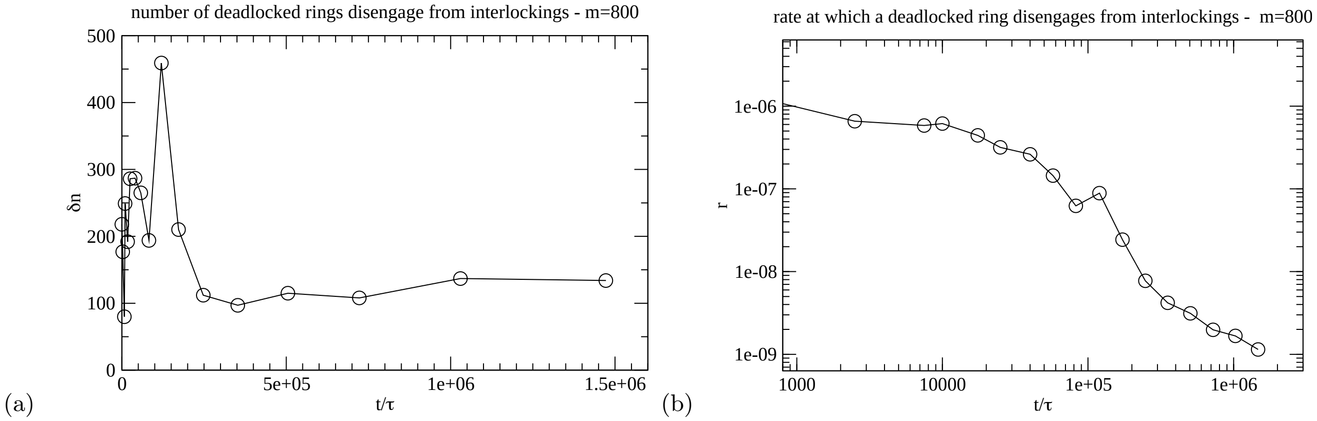

FIG. S20. (a) Number of deadlocked rings, $\delta n(t_i, t_{i+1})$ that become unraveled (non-deadlocked) between two consecutive sampled times, t_i and t_{i+1}, for $m = 800$. Notice that, due to the formation of the persistent deadlocked network of deadlocks, where deadlocked rings are typically interlocked with multiple others, the number of rings that disengage from the deadlocked clusters eventually decreases despite the sampling time intervals becoming longer and longer. The rate of disengagement per deadlocked ring, $r(t_i)$, is shown in panel (b), and was computed as $r(t_i) = \delta n(t_i, t_{i+1})/(n(t_i) \cdot (t_{i+1} - t_i))$.

The time evolution of the number of rings that thread or a threaded by other rings is shown in Fig. S21, where the populations are divided according to their deadlocked or non-deadlocked states.

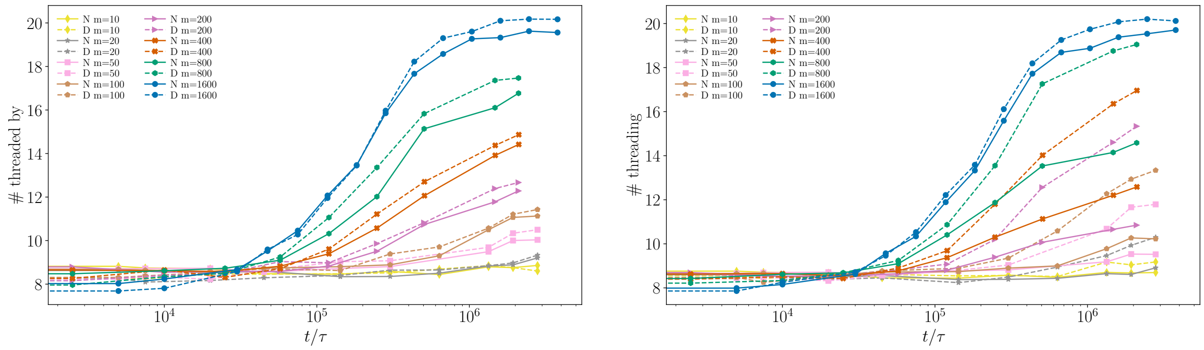

FIG. S21. Average number of rings each Nondeadlocked ("N", solid lines)/Deadlocked ("D", dashed lines) ring is threaded by/threading (left/right) for systems with different number of active chains m.

S9. GRAPH THEORETICAL ANALYSIS

The graph-theoretical analysis of the network of deadlocks for each instantaneous configuration was carried out by identifying the N rings with the nodes or vertices of the graph and their pairwise deadlocks with the node-node connections or links. The graph connectivity of each configuration was encoded in a $N \times N$ symmetric adjacency matrix, Δ, whose entries Δ_{ij} are equal to 1 if rings i and j are deadlocked, and are equal to zero otherwise.

Accordingly, the number of distinct deadlocks involving one specific ring is the degree of the corresponding node, that is the number of its connections.

Comparing increasing powers of the adjacency matrix, Δ, Δ^2, Δ^3 etc. provides information about the indirect or mediated connection between the nodes, and can thus be used to identify clusters of deadlocked rings as well as to identify cycles, see e.g. ref. [12].

The hubs of the deadlocking network were identified with various criteria, such as (i) degree centrality, corresponding to the nodes with the highest degree, and (ii) betweenness centrality, corresponding to the set of nodes with the largest percentage of shortest paths passing through them.

Schematic graph representations of the evolving network of deadlocks for $m = 800$ are given in Fig. S22.

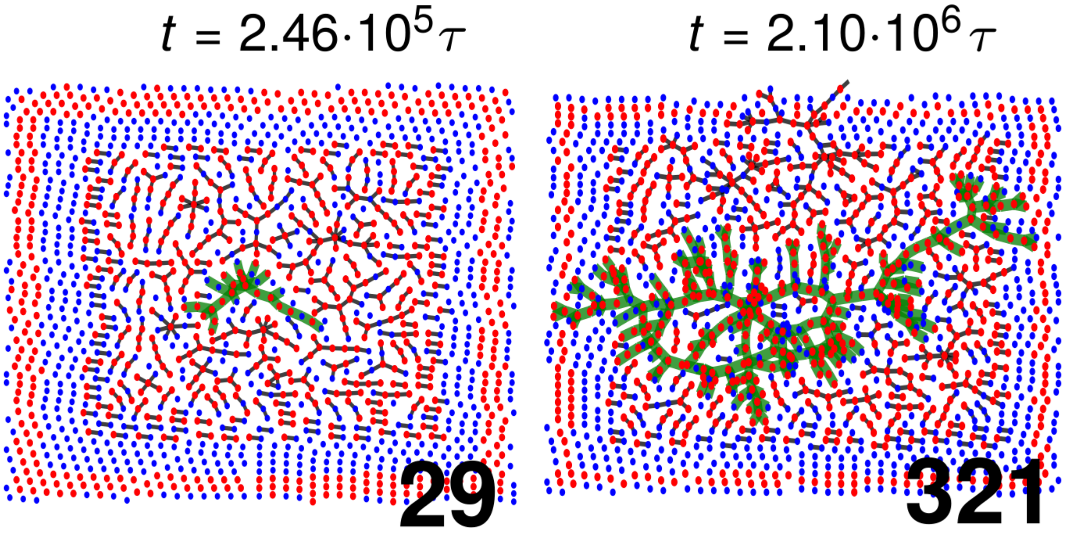

FIG. S22. Graph representations of the network of deadlocks for $m = 800$ active rings at early (left) and late (right) times. The graphs are two-dimensional abstract maps of the network of deadlocks, and hence do not represent spatial projections of the melt of rings. Red and blue nodes represent active and passive rings, respectively, while links between the nodes represent pairwise deadlocks. By convention, the largest cluster are placed at the center of the graph, and progressively smaller ones are placed more peripherally. Non-deadlocked rings thus appear as isolated nodes around the edges of the graph. The largest cluster in the sample is shown in green, and its size, i.e. the number of involved rings, is indicated in the bottom right corner. Note the presence of cycles (some relatively large) in the largest cluster of the right panel.

Fig. S23 shows the time evolution of the populations of clusters of different sizes for various activity levels.

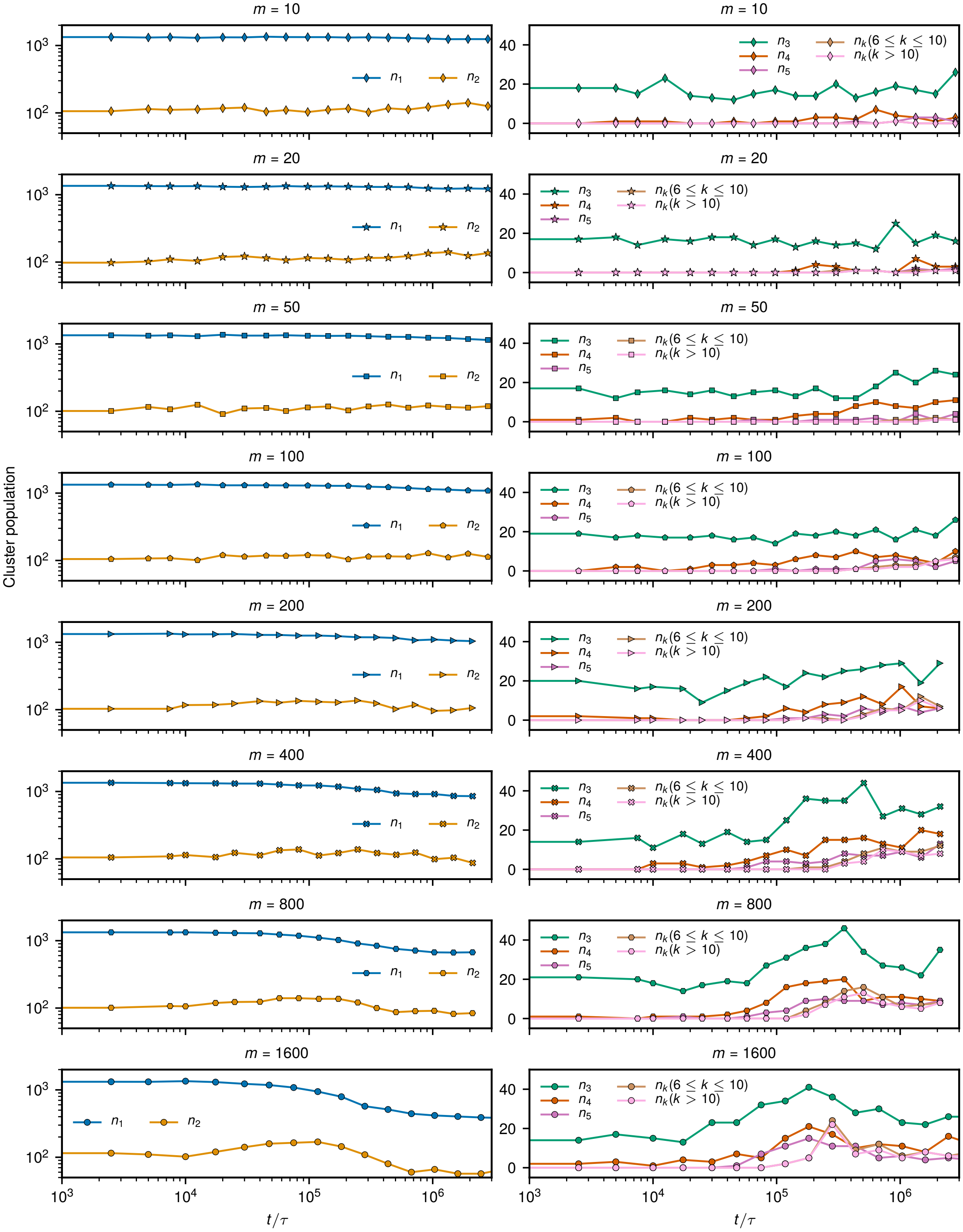

FIG. S23. Time evolution of the population of clusters of deadlocks made by k rings, n_k, for different numbers of active rings, m.

S10. TARGETED AND RANDOM RING CUTS

We assessed the response of the vitrified system to different types of targeted ring cuts in addition to random ones, all involving 25 rings. We considered a total of six different criteria for the selection of the 25 rings to be cut, hereafter indicated as c0–c5:

c0: the 25 rings with the highest degree centrality,

c1: 25 randomly chosen rings not in c0,

c2: 25 randomly chosen active rings not in c0,

c3: the 25 rings with the largest betweenness centrality,

c4: the 25 rings that optimally disconnect the deadlocks network,

c5: 25 randomly chosen rings.

The selection criterion for case c4 corresponded to the iterative search of the rings whose removal from the network most reduces the mean squared cluster size. For reference, the system evolved without cuts, too.

All ring cuts were performed by deleting the bond in the middle of the passive region. This criterion avoids interfering with the active region. Asymmetric or unbalanced cuts would instead bias the tug-of-war dynamics and elongation of the free ends of an active cut ring.

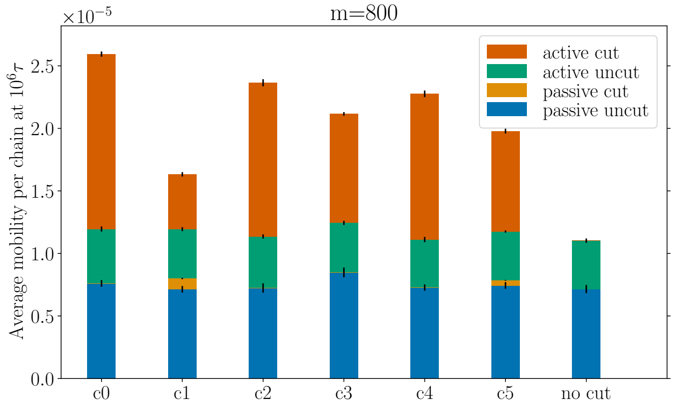

FIG. S24. Average mobility per chain after cut for the system with $m = 800$. The average mobility is computed by averaging $g_3(t)/t$ for $t \in [10^6, 1.2.10^6]\tau$, where $g_3(t)$ is the mean squared displacement reached in time t from the cut, averaged over chains. The relative contributions of the different sets of chains (active and cut, active and uncut, passive and cut, passive and uncut) to the total value are color-coded. The error bars are marked by black lines and represent the standard deviation of the $g_3(t)/t$ of the respective set.

Fig. S24 presents the average mobility per chain for the different types of cuts performed on a system of $m = 800$ active rings. Across all types, the largest mobility increment is for cut active rings. Instead, the mobility of uncut rings is practically unaffected by the cuts and is comparable to that of the reference (uncut) system. This systematic result supports the conclusion that the redundancy of the deadlock network prevents targeted/random cuts from rescuing the system from a vitrified state.

We note that sets c0, c2, c3, and c4 all included active rings, though only in c2 was this by definition. Specifically, set c0 exclusively consisted of active chains, while set c1 included 10 active and 15 passive rings, and for set c5 it was the other way around.

The mobility increment of cut rings depends strongly on the selection criterion c0–c4. The highest mobility of cut active chains is found in c0, which largely exceeds that of the control set c1. As c0 entirely consists of active chains, it is helpful to compare it to set c2, which includes only active chains by construction. The average mobility of c0 is still higher than c2, demonstrating that the key predictive feature for the large increase in mobility of set c0 is the degree centrality of the rings.

The above conclusions do not depend on the number of active rings in the system, m, as we explicitly verified by repeating the cut perturbations c0–c4 for $m = 50, 100, 200, 400$. In fact, data in Fig. S25 confirm that degree centrality (c0) is a strong predictor for increased mobility after the cuts.

Interestingly, for $m = 50$ and $m = 100$, a slight decrease in the mobility of the passive uncut rings in set c3, in contrast to the constant, or slightly increasing of the MSD is observed. Compare, for instance, the change in the blue bar of c0 to c3 across the plots in Figs. S24 and S25 for systems with larger m. This effect arguably results from the heterogeneity of the number of active chains and the active-to-passive composition across the sets; see Table S1. For instance, passive chains are typically co-opted in deadlocked clusters by active ones, and thus a smaller mobility increase is expected for small m when fewer active chains can be cut. For larger m's, the speedup can be limited by the enhanced redundancy of the network and by the fact that many active chains remain uncut. Thus, cutting only 11 or 17 active chains (c3 for systems with $m \leq 100$) does not free the passive uncut chains as much as cutting 21 or 23 active chains (c0 for $m \leq 100$). The same holds for $m \geq 200$, but in this case, the passive uncut chains in c0 are already vitrified by the redundant network of deadlocks.

m	c0	c1	c2	c3	c4	c5
50	21/4	0/25	25/0	11/14	16/9	-
100	23/2	1/24	25/0	17/8	20/5	-
200	23/2	4/21	25/0	17/8	19/6	-
400	24/1	4/21	25/0	16/9	24/1	-
800	25/0	10/15	25/0	25/0	25/0	15/10

TABLE S1. Ratio of the number of the cut active and the number of the cut passive chains in the set of the various cut types.

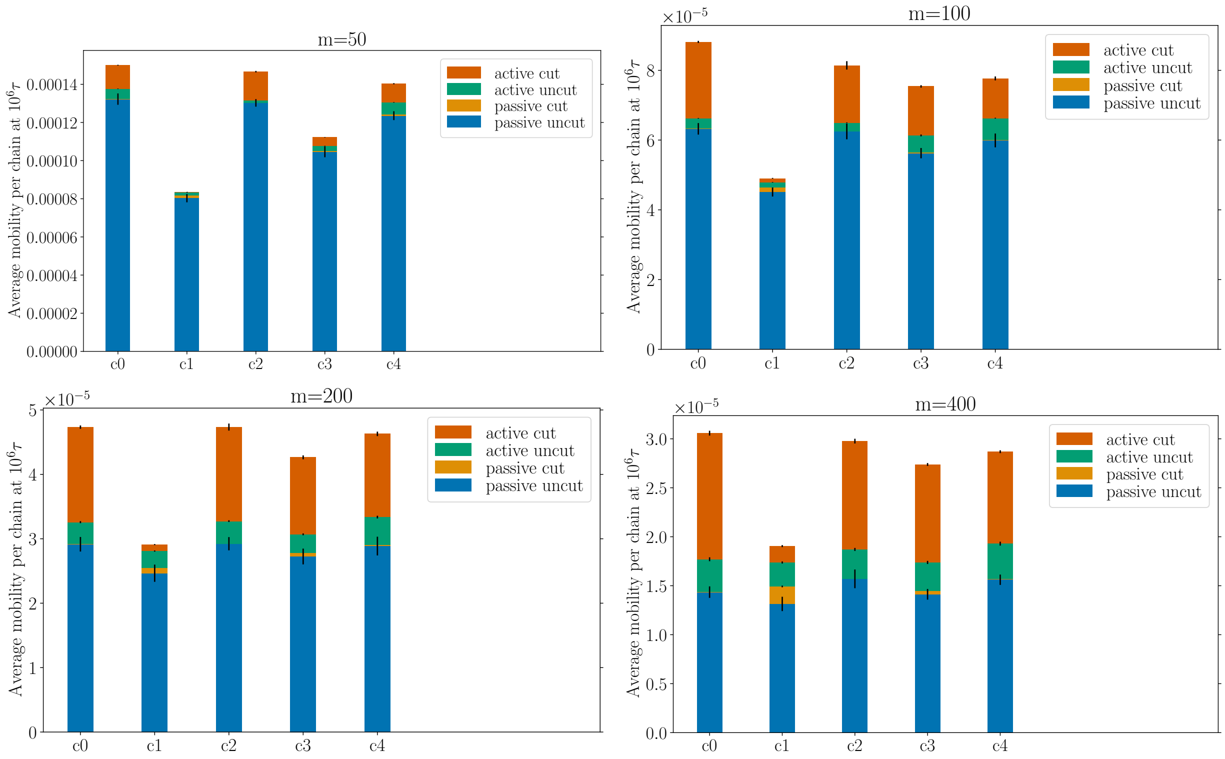

FIG. S25. Average mobility per chain after cut for systems with $m = 50$ to $m = 400$. The average mobility is computed by averaging $g_3(t)/t$ for $t \in [10^6, 1.2.10^6]\tau$, where $g_3(t)$ is the mean squared displacement reached in time t from the cut, averaged over chains. The relative contributions of the different chain sets (active and cut, active and uncut, passive and cut, passive and uncut) to the total value are color-coded. The error bars are marked by black lines and represent the standard deviation of the $g_3(t)/t$ of the respective set.

[1] Kurt Kremer and Gary S. Grest. Dynamics of entangled linear polymer melts: A molecular-dynamics simulation. *J. Chem. Phys.*, 92(8):5057–5086, 1990.

[2] Ralf Everaers, Hossein Ali Karimi-Varzaneh, Frank Fleck, Nils Hojdis, and Carsten Svaneborg. Kremer–grest models for commodity polymer melts: Linking theory, experiment, and simulation at the kuhn scale. *Macromolecules*, 53(6):1901–1916, 2020.

[3] Steve Plimpton. Fast parallel algorithms for short-range molecular dynamics. *J. Comput. Phys.*, 117(1):1–19, 1995. `http://lammps.sandia.gov`.

[4] Jan Smrek, Iurii Chubak, Christos N. Likos, and Kurt Kremer. Active topological glass. *Nat. Commun.*, 11(1):26, 2020.

[5] Alexandra Zidovska, David A. Weitz, and Timothy J. Mitchison. Micron-scale coherence in interphase chromatin dynamics. *Proc. Natl. Acad. Sci.*, 110(39):15555–15560, 2013.

[6] Robijn Bruinsma, Alexander Y. Grosberg, Yitzhak Rabin, and Alexandra Zidovska. Chromatin hydrodynamics. *Biophys. J.*, 106(9):1871–1881, 2014.

[7] Jan Smrek and Alexander Y. Grosberg. Minimal surfaces on unconcatenated polymer rings in melt. *ACS Macro Lett.*, 5(6):750–754, 2016.

[8] Jonathan D. Halverson, Won Bo Lee, Gary S. Grest, Alexander Y. Grosberg, and Kurt Kremer. Molecular dynamics simulation study of nonconcatenated ring polymers in a melt. II. Dynamics. *J. Chem. Phys.*, 134:204905, 2011.

[9] Jonathan D. Halverson, Won Bo Lee, Gary S. Grest, Alexander Y. Grosberg, and Kurt Kremer. Molecular dynamics simulation study of nonconcatenated ring polymers in a melt. I. Statics. *J. Chem. Phys.*, 134:204904, 2011.

[10] C. C. Adams. *The Knot Book*. Freeman, 1994.

[11] Jan Smrek, Kurt Kremer, and Angelo Rosa. Threading of unconcatenated ring polymers at high concentrations: Double-folded vs time-equilibrated structures. *ACS Macro Lett.*, 8:155–160, 2019.

[12] Enzo Orlandini and Cristian Micheletti. Topological and physical links in soft matter systems. *J. Phys.: Condens. Matter*, 34(1):013002, 2022.
